# Identification of factors associated with acute malnutrition in children under 5 years and forecasting future prevalence: assessing the potential of statistical and machine learning methods

**DOI:** 10.1136/bmjph-2024-001460

**Published:** 2025-03-04

**Authors:** Meike Reusken, Christopher Coffey, Frans Cruijssen, Bertrand Melenberg, Cascha van Wanrooij

**Affiliations:** 1 Department of Econometrics and Operations Research, Zero Hunger Lab, Tilburg University, Tilburg, Noord-Brabant, Netherlands; 2 Operations Research and Logistics, Wageningen University & Research, Wageningen, Gelderland, Netherlands; 3 World Food Programme, Rome, Italy; 4 Department of Econometrics and Operations Research, Tilburg University, Tilburg, Noord-Brabant, Netherlands

**Keywords:** Nutrition Surveys, Public Health Practice, methods

## Abstract

**Introduction:**

Eliminating acute malnutrition in children under 5 years of age stands as a critical health priority outlined in the United Nations Sustainable Development Goal 2, ‘Zero Hunger’. This requires targeted provision of treatment and preventative services. However, accurately forecasting future prevalence of cases remains challenging, with the application of predictive models being notably scarce. Addressing this gap, this paper aims to identify factors associated with Global Acute Malnutrition (GAM) and explores the potential of machine learning in predicting its prevalence using data from Somalia.

**Methods:**

Survey data on GAM prevalence systematically collected in Somalia every 6 months at a district level from 2017 to 2021 were collated alongside a range of potential climatic, demographic, disease, environmental, conflict and food security-related factors over a matching time period. We conducted both simple and multiple, parametric and non-parametric statistical analyses to identify factors associated with GAM to be used as input in forecasting future GAM prevalence. We then applied tree-based machine learning algorithms to a dataset comprising the GAM prevalence estimates and associated factors to try to forecast the trajectory and fluctuations in GAM prevalence 6 months into the future.

**Results:**

We found factors statistically associated with GAM prevalence relating to rainfall, land vegetation quality, food security status, crop production and demographics. The majority of these associations were nonlinear, motivating the use of tree-based machine learning–based forecasts. Among the forecasting methods tested, random forest machine learning proves to be the most effective and was found to accurately forecast the direction of GAM prevalence in test data for many of the districts in Somalia.

**WHAT IS ALREADY KNOWN ON THIS TOPIC**

**WHAT THIS STUDY ADDS**

- We find factors related to rainfall, land vegetation quality, food security status, crop production and demographics to be statistically associated, generally in nonlinear fashion, with acute malnutrition in Somalia.
- Tree-based machine learning predictions are effective in forecasting directions and absolute changes in the prevalence of acute malnutrition for a large majority of Somalia districts over a 6-month period.

**HOW THIS STUDY MIGHT AFFECT RESEARCH, PRACTICE OR POLICY**

- Governmental and non-governmental organisations can leverage machine learning methods, which appear promising for improved forecasting within early warning systems, to enhance planning and anticipatory action.
- To increase the accuracy of the presented methods further, additional potentially predictive factors should be explored.
- Improving the frequency, completeness and quality of data on acute malnutrition and associated factors can further improve model performance.

## Introduction

 Eliminating acute malnutrition is a major global health priority that is central to the United Nations’ Sustainable Development Goals and is directly addressed under Goal 2 (‘Zero hunger’), focusing on ending hunger, achieving food security and promoting sustainable agriculture.^1^ Global acute malnutrition (GAM), including both moderate and severe forms, is a condition in children under 5 years of age characterised by nutritional oedema, that is, extracellular water in the body, and/or child wasting, that is, being too thin for their height due to weight loss or the failure to gain weight resulting in low weight-for-height and/or a low mid-upper arm circumference.^2^ While oedema is rare and tends to be most common in tropical areas and in children with serious complications, child wasting is more common. In 2022, 45 million children under 5 years of age were estimated to suffer from child wasting, which corresponds to 6.8% of all children in that age group at the time.^3 4^ Each year, 800 000 deaths are attributable to child wasting, and this figure is likely underestimated.^5^

Immediate causes include weight loss due to infections and dietary factors including suboptimal breastfeeding and complementary feeding practices and acute and chronic food insecurity, although these have been found to vary by context. Underlying causes include social and environmental factors such as poverty, poor education, healthcare access and contaminated environments, as well as conflicts and climate shocks.^6^

Programming to tackle acute malnutrition encompasses provision of services for both treatment and prevention. To adequately plan for programming, it is necessary to estimate how many cases to expect, when and where. The standard approach to estimate the yearly cases of GAM is to multiply the GAM prevalence estimates from cross-sectional surveys by an incidence correction factor (ICF), typically of 1.6.^7^ However, research has shown that this ICF consistently underestimates the actual number of cases of GAM.^8^,^12^ Proposals for more accurate ICFs have been suggested, which make use of additional data sources like programme admissions in the same geographic area of the prevalence survey and programme coverage estimates.^8 11^ Improved context-specific estimation methods for predicting both GAM incidence and prevalence in both current and future time periods are vital to inform efforts for both prevention and treatment.

Our contribution involves collecting a set of publicly available input variables and analysing and leveraging their relationship with GAM prevalence in two steps. First, we conduct a statistical relationship analysis to investigate the significance and nature (linear vs non-linear) of the relationship. This exploration is prompted by discussions with practitioners working with children affected by GAM, who highlighted the potential for nonlinear, threshold-like relationships between potential factors and acute malnutrition. In the second step, we incorporate the identified associated factors into tree-based machine learning algorithms to forecast the prevalence of GAM.

Recent literature on forecasting food security indicators demonstrates the potential of advanced statistical and machine learning models and the use of diverse data sources to provide accurate input for policy responses. For example, a regression model was proposed that used market prices, weather and demographic data to forecast food insecurity status in Malawi, successfully identifying 83 to 99% of the most food-insecure areas.^13^ As another example, a statistical framework was used to effectively forecast transitions in food insecurity status, based on key predictors such as agronomic, weather, conflict and economic variables.^14^ Also, machine learning techniques were used to forecast food security transitions in Ethiopia, using predictors like food security history, surface soil moisture, food prices and conflict data, with a gradient boosting model.^15^ Finally, the effectiveness of machine learning in predicting food insecurity across villages in three sub-Saharan African countries was demonstrated using data on market prices, household assets and climate. The study advocates collaboration between modellers and users—that is, policymakers and practitioners—to ensure the inclusion of relevant drivers, proper error handling and good contextual understanding.^16^

Our research narrows its focus to specifically forecasting GAM, a key indicator of child health within the context of food security. To the best of our knowledge, only four studies have contributed similar perspectives,^17^,^20^ also concentrating on predicting GAM or closely related child health indicators. We compare these studies with our research in table 1, detailing the organisation involved (row 2), the country from which data originates (row 3), the time span of the data (row 4), the model used for forecasting (row 5), the variable being predicted (row 6), the variables used as input (row 7), the horizon predicted ahead in time (row 8) and the conclusions (row 9). The table shows that our research differs from these previous studies in several respects. Specifically, our research’s contribution can be summarised as follows: (1) we use a large and the most recent set of publicly accessible data for the country of Somalia; (2) we conduct extensive analyses of statistical relationships to identify factors associated with GAM and gain insights into the nature of these relationships, prior to forecasting, to avoid potentially irrelevant variables and incorrect model assumptions that could increase the risk of model overfitting (which may lead to a decrease in forecasting performance); and (3) we compute forecasts for 6 months ahead, being among the farthest ahead in time, in order to be able to use results effectively in practice, for example in early warning systems.

**Table 1:** Overview of literature related to forecasting acute malnutrition

1:	Study 1^17^	Study 2^18^	Study 3^19^	Study 4^20^	This study
2: Organisation involved	–	–	United Nations Children’s Fund	Armed Conflict Location & Event Data Project	United Nations World Food Programme
3: Country	Kenya	Bangladesh, Ethiopia, Ghana, Guatemala, Honduras, Kenya, Mali, Nepal, Nigeria, Senegal, Uganda	Somalia, South Sudan	36 Sub-Saharan countries	Somalia
4: Years	2000–2005	2004–2016	2014–2018	2003–2019	2017–2021
5: Model	Linear models (reduced-form dynamic models)	Random forest	Generalised linear models, random forest	Random forest	Tree-based machine learning
6: Target variable	Children’s mid-upper arm circumference (MUAC)	Asset poverty, child stunting, child wasting, healthy weight, underweight women	Prevalence of GAM and SAM	Prevalence of GAM	Prevalence of GAM
7: Input variables	Herd size, lactation rates, mortality rates, off-take rates, climate and forage conditions, historical levels of child MUAC	Location/remoteness, meteorological, vegetative, market, food price, conflict data	Livelihood data, disease incidence, vegetation index, water price, food distribution, rainfall, terms of trade, conflict incidence, humanitarian data	Rainfall, temperature, vegetation, lethal and non-lethal conflict events, population, night-time light intensity and distance to capital city	Historic levels of GAM, cases of conflict, IPC, crop production, land vegetation quality, rainfall, food security-related factors and demographics
8: Prediction horizon	1 month and 3 months ahead	Contemporaneous	Contemporaneous	1 month to 12 months ahead	6 months ahead
9:Conclusions	It is possible to generate accurate forecasts of child MUAC, a key indicator of human welfare. The study emphasises the need for improved methods and standardised data collection procedures.	Child wasting can be predicted with modest accuracy, especially when jointly predicted with other poverty and malnutrition indicators. While machine learning is more cost-effective, it is not a complete substitute for traditional survey methods in informing development efforts.	Predictive models exhibit low performance, rendering them unsuitable for immediate action. Further studies are recommended, particularly with increased data, given the potential of random forest prediction.	Predictions exhibit strong predictive performance and remain stable across the different prediction horizons. Data availability and granularity pose challenges in this context. Framework is promising as a complement to traditional early warning systems.	Factors associated with GAM are identified, with many relationships being nonlinear. Leveraging this understanding, forecasts of GAM using machine learning models show promising results, which can facilitate anticipatory planning for treatment and prevention.

GAM, global acute malnutrition; IPC, Integrated Food Security Phase Classification; SAM, severe acute malnutrition.

## Methods

### Patient and public involvement

This study was commissioned by the World Food Programme (WFP) and was conducted in close cooperation with the WFP Somalia Country Office, Regional Bureau Nairobi and Head Quarters staff.

### Data

After assessing data availability in several countries in which the WFP operates programmes for the management of acute malnutrition, Somalia was deemed a good candidate country.

Somalia is divided into 18 regions, and these are in turn subdivided into 74 districts.^21^ Historic data on the prevalence of GAM were obtained through the publicly available database of population-based surveys collected through the Somalia Food Security and Nutritional Analysis Unit (FSNAU) (for more details of the FSNAU and their collection methods, see ^22^). The data contain populations-based survey estimates of GAM among children aged 6–59 months, collected after the Gu (April–June) and Deyr (October–December) rainy seasons, resulting in two yearly observations, one in January and one in July. The prevalence of GAM is based on the WHO standards for GAM by weight-for-height, which involves comparing a child’s weight-for-height measurements to reference growth standards. This comparison gives a Z-score that indicates how many standard deviations the child’s measurements are from the median of the reference group. The presence of oedema or a weight-for-height Z-score below −2 constitutes GAM.^23^ The average prevalence across all the districts in Somalia for July 2021 was 13.8% among the population under 5 years of age. If 10% or more of the children are classified as suffering from GAM, this is generally considered to be a serious emergency, and with over 15%, the emergency is considered critical (IPC Global Partners, p.14).^24^

In 2022, levels of acute malnutrition in Somalia were classified as high by the Joint Malnutrition Estimates published by the WHO and UNICEF.^4^ Seasonal and shock-induced spikes in prevalence are common in the country, and levels in some areas of the country are consistently above the 15% threshold for a critical emergency.^22^

Data on any potential factor associated with GAM were collected from publicly available sources, including variables related to conflicts, disease, rainfall, vegetation, crop production, food prices, COVID-19, population demographics and Integrated Food Security Phase Classification (IPC) phases. The IPC provides a composite indicator which integrates several sources of information relevant to food insecurity. The indicator is scored between one to five, where one represents minimal food insecurity and five represents famine conditions.^25^ In the interest of reproducibility and transparency, a comprehensive guide on how to access the consulted databases can be found in section 1 of the online supplementary material.

For our case study, we selected a geographic area and time based on data availability, resulting in the inclusion of all 74 districts and a time span from July 2017 to July 2021 (totals 74×49 = 3626 monthly time periods and 74×9 = 666 biannual time periods). Since some observations of GAM prevalence were missing within this time period, several data points were discarded. Specifically, for the statistical relationship analysis, conducted with monthly observations, only the missing data points were excluded, resulting in 3584 monthly time periods. For the forecasting analysis, conducted with biannual observations, all districts lacking at least one of the biannual observations were excluded from the analysis, leaving 65 districts and nine distinct time points, resulting in a total of 585 biannual observations (i.e., 65×9 = 585).

Initial examination of the input variables regarding the number of missing observations and reliability resulted in the exclusion of the number of COVID-19 cases, measles cases, acute watery diarrhoea/cholera cases and deaths, malaria cases and prices of water, sorghum, maize and red rice. The variables crop diversity and crop production contained observations for only 46 districts, and we created observations for the missing districts by spatially interpolating values based on the surrounding districts. The resulting variables are examined for correlations with each other. When highly correlated variables (correlation coefficient >0.9) were identified, all but one of the correlated variables were removed. This led to omitting the population count of children under the age of five, due to its almost perfect correlation with the total population count.

The resulting selection of data is summarised in table 2, in which the first two columns provide the variable name and a brief description, while the number of observations available in the data, per year, is given in column 3. The mean (*µ*) and standard deviation (*σ*), calculated on the basis of the available data, are given in column 4, and column 5 presents the organisation from which the data was sourced. All the variables contain data at the district level within Somalia. Scatter plots of each selected variable against GAM can be found in section 2 of the online supplementary material.

**Table 2:** Summary of variables used in the analysis

Variable	Description	Yearly observations	(*µ*, *σ*)	Source
GAM	Prevalence of GAM as percentage of under-five population	2	(14.5, 3.8)	FSNAU*
Conflicts	Total number of conflicts such as armed battles, explosions, protests and riots	12	(5.6, 9.1)	Armed Conflict Location and EventData†
Rainfall	Rainfall (millimetres) as a deviation from the district monthly mean	12	(−1.2, 25.1)	FSNAU
NDVI	Normalised Difference Vegetation Index, a metric for quantifying the land vegetation quality	12	(0.3, 0.1)	FSNAU
IPC	The percentage of the population that is in IPC phase 3 or higher	2	(0.14, 0.11)	IPC‡
Crop diversity	A count of the different types of crops being produced	2	(2.1, 1.6)	FSNAU
Crop production	Crop production in metric tons divided by the area	2	(434.4, 658.8)	FSNAU
Population	Population count	2	(167 638.4, 253 802.4)	IPC
Density	The population count divided by the area	2	(147.5, 1059.5)	IPC and World Bank§

All web pages are last accessed on 16 February 2024.

* See https://fsnau.org/.

† See https://acleddata.com/data-export-tool/.

‡ See https://www.ipcinfo.org/.

§ See https://microdata.worldbank.org/index.php/catalog/3811.

FSNAU, Somalia Food Security and Nutritional Analysis Unit; GAM, global acute malnutrition; IPC, Integrated Food Phase Classification; NDVI, normalised difference vegetation index.

To ensure fair comparison among the relative effects of the variables, data are normalised, which involves scaling the data by subtracting the variable mean and dividing by the standard deviation. For the variables containing only 2 yearly observations, linear interpolation is used to increase the number of observations, which is needed for the statistical relationship analysis conducted at the monthly observation interval. Conversely, for the forecasting analysis, which is conducted at the biannual observation interval, averaging is used to reduce the number of observations for conflicts, rainfall and normalised difference vegetation index (NDVI). Furthermore, for each of the variables in table 2, time-lagged derived variants are introduced to account for time delays in the effects. Specifically, for the statistical relationship analysis, a 1-month lag is created along with either an average of the preceding 3 months (for NDVI) or a sum of the preceding 3 months (for conflicts and rainfall). For the other variables, with only two yearly observations, we generate a 6-month lag. In the forecasting analysis, 6-month lags are used for all variables. These preprocessing steps of averaging and creating derived variants, along with occasional discarded observations due to missing values in input variables, further reduce the number of time periods available for analysis. Specifically, this narrows down to 449 biannual observations and 3070 to 3081 monthly observations, depending on the type of derived variant used.

The original variable for GAM serves as the target variable in our study, while all other variables (including the derived variants) as well as the 6-month lag of GAM are input variables.

### Statistical relationship analysis: identifying risk factors

The preprocessed variables are statistically analysed in a three-step procedure to study whether and how the input variables are related to the target variable, GAM. The first two steps involve analysing a single input variable at a time, while the third step involves considering multiple input variables simultaneously.

#### Simple linear regression

The first step investigates the impact of each input variable individually on GAM. In this context, we establish the significance of the relationship, that is, whether the change in one input variable is associated with a change in GAM. This question can be answered using simple linear regression and studying the significance of the relationship. We use the significance levels to classify the resulting association into four levels, ranging from a ‘very low’ association to a ‘very high’ association.

#### Simple non-parametric regression

In the second step, we determine the nature of the univariate relationship, that is, understanding how an input variable is linked to GAM. To accomplish this, we examine for each input variable separately whether the link with GAM can be described by a simple linear relationship. To investigate this, we estimate the link between GAM and each input variable non-parametrically and check whether a linear relationship, estimated using ordinary least squares, fits inside the non-parametrically estimated 95% uniform confidence band (Hardle W, Theorem 4.3.1).^26^ Such a confidence band establishes a range within which the true link between GAM and the input variable is likely to lie with 95% confidence. In the non-parametric approach, we use a commonly applied kernel regression, with the quartic kernel.^26^ The bandwidth *h_n_*, depending on the sample size *n*, controls the smoothness of the estimated regression function. A smaller bandwidth results in a more sensitive fit to the data, capturing finer details, while a larger bandwidth produces a smoother fit, potentially missing local variations. *h_n_* is determined through a combination of visual assessment and the standard rule of thumb (ie, $h_{n}\;=\;1.06\hat{\sigma }n^{-1/5}$, with $\hat{\sigma }$ the estimated standard deviation of the input variable). We categorise the outcomes as *linear* if the linear regression band fits inside the non-parametric 95% uniform confidence band (Since non-parametric regression converges more slowly than linear regression, the formal rule based on asymptotics is to check whether the linear regression curve fits within the non-parametric confidence bands. However, in our finite sample case, we apply a stricter version of this rule by requiring the linear confidence bands, rather than just the linear curve, to be within the nonparametric confidence bands.) and *nonlinear* in all other cases. This analysis is conducted at the regional level to allow the link to be region-specific, thereby accounting for heterogeneity. When a linear relationship was identified in at least 50% of the regions, we indicate the link between an input variable and GAM as being linear.

#### Multiple linear regression

In the final step, our focus is on finding the input variables that collectively explain developments in GAM, that is, which variables are factors associated with GAM? This step of the statistical relationship analysis checks the presence of a statistically significant relationship using multiple linear regression models. We use several models to ensure that at most one variant of the same input variable enters the regression equation, to avoid multicollinearity problems. Specifically, we tested three models, each considering different variants of the input variables, with at most one variant of each variable present: model 1 considers the original variants; model 2 includes either a 6-month or 1-month lag; and model 3 includes either a 6-month lag, the sum of the preceding 3 months or the average of the preceding 3 months. Additionally, each of these regression models includes dummy variables to consider the effect of months and regions. We analyse the regression estimates and the standard errors and classify the association of the input variables with GAM again into four levels, ranging from a ‘very low’ association to a ‘very high’ association. Lastly, we make use of F-tests to classify the association with GAM into four levels of the dummy variables for months collectively, as well as the dummy variables for regions collectively. We calculated the (asymptotic) standard errors assuming the standard linear regression assumptions and allowing for heteroskedasticity and autocorrelation, using Newey-West standard errors.^27^

### Tree-based machine learning: predicting directions and changes

Decision trees are useful methods for non-parametric modelling.^28^ Non-parametric modelling means that no prior assumptions on the distribution of the underlying data (such as linearity) are imposed and that the nature of the relationship is inferred based on the data. Decision trees work by identifying cut-off points in the data that predict changes in the target variable. For example, they could identify that GAM increases when there has been a period of drought of *α* months. The tree structure, including such cut-off values for *α*, is constructed through a learning process that selects features and thresholds to minimise forecasting errors.^28^ Tree-based machine learning is the overarching term for the individual decision tree as well as tree ensembles, which combine multiple decision trees to arrive at a more accurate prediction.^29^

We focus on tree-based machine learning methods because their non-parametric character makes them especially suitable when the statistical relationship analysis identifies nonlinearities, which is the case for our data. Furthermore, they are able to capture threshold-like relationships and tipping points within the data where input variables cross certain thresholds. Lastly, tree-based machine learning methods have shown outstanding forecasting accuracy, also in the field of health.^30^ ^31^ From the range of tree-based machine learning techniques, we concentrate on gradient boosting^32^ and random forest,^33^ as they represent the two most commonly employed methods and are known for their high performance.

Using these two tree-based methods, we generate forecasts for GAM at the district level, predicting outcomes 6 months in advance. As outcomes, we forecast the changes between time periods, and we evaluate the performance of this forecast in two ways. First, we identify whether the model is correct in predicting an increase or decrease in GAM. Second, we evaluate the capability of the model to forecast the change in GAM in absolute terms.

To produce these forecasts, we start by selecting the preprocessed biannual variant from our target variable ‘GAM’, as well as the input variables that are found to be significantly related to GAM in the preceding statistical relationship analysis (an established correlation in either the simple or multiple regression ensures that the input variable is considered). To secure that data are available at the time of the forecast (and no future data is used), we only use the derived 6-month lags of the input variables. We furthermore apply cross-validation, a statistical technique used for hyperparameter tuning and assessing the performance and generalisability of a model by partitioning the dataset into subsets: training, validation and test sets. To guarantee that the training set consists only of observations that occurred prior to the observation that forms the validation or test set, we make use of the time-series variant of cross-validation (with one train-test split and two splits for hyperparameter tuning) (Montgomery D C, et al., section 5.10). Specifically, we use the first *β* time periods to train our model (training set), the next time period to try out different model configurations (validation set) and the last two time periods to assess the models’ predictive capabilities (test set). We apply this splitting procedure for two values of *β* and average the prediction results. To explore multiple configurations, we employ grid search to tune commonly varied hyperparameters. In the case of random forest, we optimise the number of features, the number of trees and the number of samples required to split an internal node. Additionally, for gradient boosting, we tune the number of features, the number of trees, the number of samples required to split an internal node, the tree depth and the learning rate. For more details on the setup for hyperparameter tuning, see section 3 of the online supplementary material.

To the best of our knowledge, no benchmark solutions currently exist for forecasting GAM prevalence 6 months ahead. Existing methods related to this task typically target different target variables and different horizons predicted ahead in time (cf. table 1). Consequently, we employ two widely used simple benchmarks in forecasting analysis: simple exponential smoothing, which calculates a weighted average over the preceding forecast and the actual value in the previous period, and naive forecasting, which simply uses the actual value from the previous period as the forecast.^34^ Forecast *F_t_*_+1_ based on simple exponential smoothing is equal to *αY_t_* + (1 − *α*)*F_t_* with *F_t_* denoting the previous forecast, *Y_t_* denoting the actual value in the previous period and *α* ∈ [0*,* 1] denoting the smoothing parameter. The naive method is the same as simple exponential smoothing for *α*=1.

## Results

All results were obtained using Python 3.9, with the Python libraries statsmodels for regression analyses and exponential smoothing and scikit-learn for machine learning models.

### Factors associated with global acute malnutrition (GAM)

Table 3 summarises the main findings of the statistical relationship analysis. Column 1 lists the names and variants of the input variables, while column 2 reports results regarding the statistical significance of the relationship with GAM for each input variable separately. Column 3 presents the result for the nature of each relationship. Finally, column 4 reports the findings regarding the statistical significance when conditioning on input variables collectively. The regression results identify statistical associations which we classify using the following *p* value thresholds:

‘very high’: p≤0.01;

‘high’: 0.01§amp;lt;p≤0.05;

‘low’: 0.05§amp;lt;p≤0.3;

‘very low’: p§amp;gt;0.3.

**Table 3:** Results of the statistical relationship analysis

Input variable	Simple linear regression(very high, high, low, very low)	Simple non-parametric regression(linear, non-linear)	Multiple linear regression(very high, high, low, very low)
GAM			
6lag	very high	linear	very high
Conflicts			
0lag	very high	nonlinear	very low
1lag	very high	nonlinear	very low
3sum	very high	nonlinear	low
Rainfall			
0lag	very high	nonlinear	high
1lag	high	nonlinear	very low
3sum	low	nonlinear	very low
NDVI			
0lag	very high	linear	high
1lag	very high	linear	very high
3avg	very high	linear	very high
IPC			
0lag	very high	nonlinear	very high
6lag	very high	nonlinear	very low
Crop diversity			
0lag	very high	nonlinear	very high
6lag	very high	nonlinear	very high
Crop production			
0lag	very high	nonlinear	low
6lag	very high	nonlinear	high
Population			
0lag	very high	nonlinear	low
6lag	high	nonlinear	high
Density			
0lag	very high	nonlinear	very low
6lag	very high	nonlinear	high
Regions (18)	very high (12), high (0), low (4), very low (2)	–	very high
Months (12)	very high (0), high (3), low (3), very low (6)	–	high

We use the following abbreviations: 0lag for a zero-month lag, that is, the original variant; 1lag for the 1 month lag; 6lag for the 6 month lag; 3sum for the sum of the preceding 3 months; and 3avg for the average of the preceding 3 months. For the dummy variables representing the 18 regions and 12 months, the table reports in column 2, in brackets, the count of dummies. This table shows the multiple linear regression results for the (asymptotic) standard errors calculated assuming the standard linear regression assumptions, when allowing for heteroskedasticity and autocorrelation we obtain closely resembling results, see section 4 of the online supplemental material.

The regression coefficients and significance levels related to regressions underlying the findings in table 3 are available in section 4 of the online supplemental material.

The results from the statistical relationship analysis reveal that the various variants of the same input variable exhibit very similar association effects on GAM, especially for the simple linear regression results. In this setting, the established correlations are consistent for all variants of the same input variable, except for the input variable ‘rainfall’, for which only two out of the three variants show a high or very high association.

The results show that the 6-month lag of GAM is very highly associated with GAM itself, as an individual correlation in isolation as well as in the presence of the other input variables. This relationship is characterised as linear.

Additionally, we observe that, aside from conflicts, all other input variables are identified as factors associated highly or very highly with GAM as well, in at least one of the multiple regression variants, that is, even when other input variables are included in the model. Notably, the majority of all relationships are nonlinear in nature.

For the input variable ‘conflicts’, which shows a low or even very low association with GAM in the multiple linear regression, a very high association is still found in isolation from other input variables. The fact that there is a (very) high association in the simple regression, while not so in the multiple regression, may be attributable to ‘conflicts’ capturing the effect of other (not included) factors associated with GAM in the simple regression.

The simple linear regression results for the region dummies indicate that there are 12 regions for which the association with GAM is very high. For the dummy variables for January, July and August, we find a high association. In case of the other months, the association is low or even very low. The results of the multiple linear regression, in which we condition on other input variables, show that GAM is indeed highly associated with time and very highly associated with regions.

The outcomes of this section provide a strong motivation to proceed with the next step, that is, to forecast GAM trends using tree-based machine learning approaches that can deal with nonlinearities in a flexible way.

### Forecasts of child wasting trends

We present results on the relative performance of the tree-based machine learning predictions gradient boosting and random forest compared with two benchmarks: simple exponential smoothing and naive forecasting. We look at four performance criteria: (1) overall mean absolute error (MAE), (2) ability to correctly forecast the direction of change, (3) ability to accurately forecast the absolute value of change and (4) ability to provide reliable granular forecasts on the level of districts.

The random forest prediction, with a MAE of 0.502, demonstrates the best performance in forecasting the target variable, GAM, 6 months into the future. It slightly surpasses gradient boosting (MAE=0.551) and the simple exponential smoothing benchmark (MAE=0.562). It considerably outperforms the naive (MAE=1.004) benchmark.

**Figure 1:**
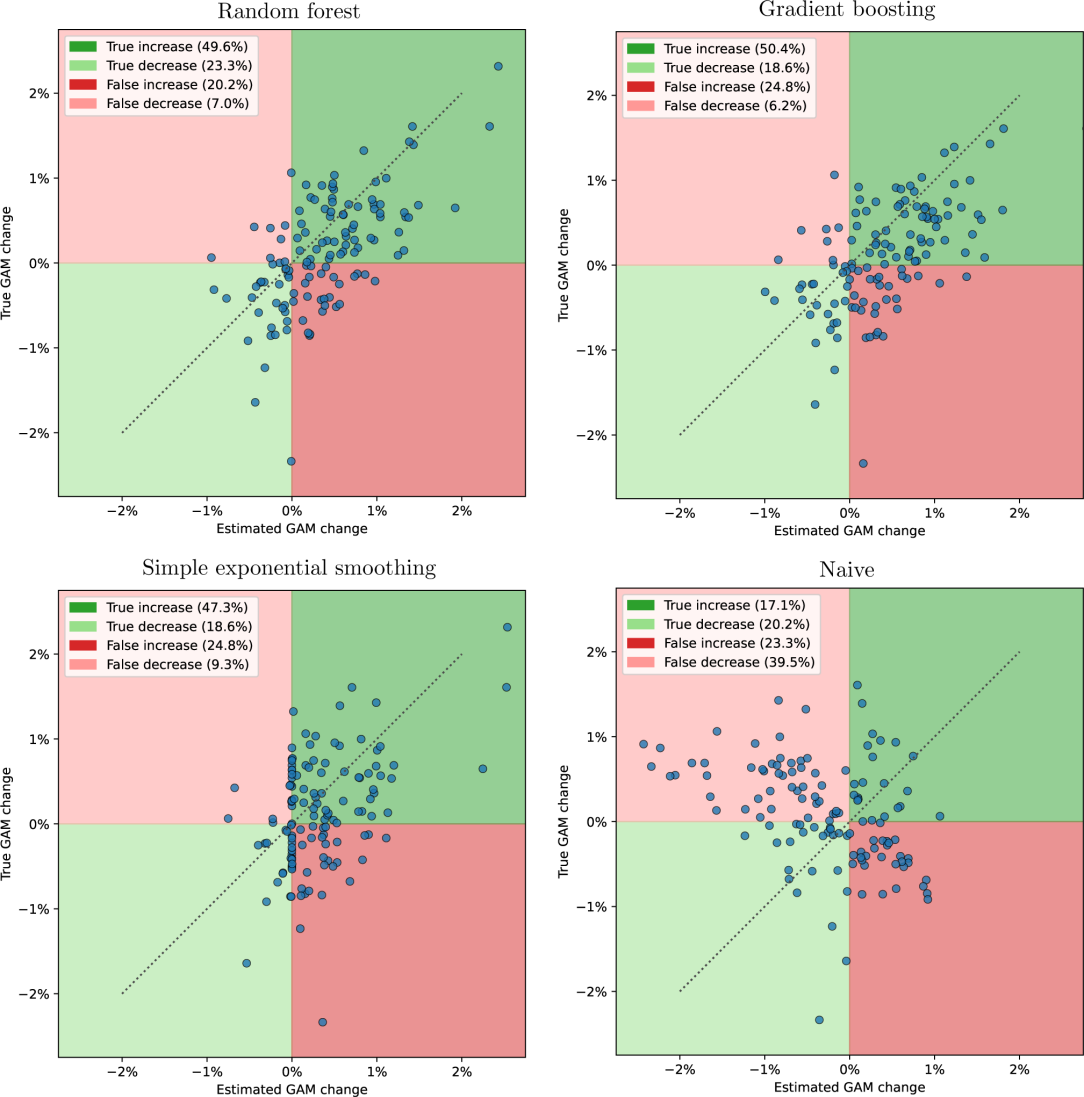
Scatter plots showing the forecasted and actual change in global acute malnutrition (GAM), with the axes indicating the change in percentage points. There are three test set observations from the districts of Belet Weyne, Bulo Burto and Jalalaqsi, where the true GAM change is approximately −2.65%. These observations fall outside the boundaries of the plots and are therefore not shown. For these three observations, we arrive at a false increase prediction.

Providing more details regarding their performance, figure 1 presents results related to the quality of our forecasts from two additional perspectives. First, it presents the predicted direction of GAM, that is, whether we forecast an increase (ie, x-axis greater than zero) or decrease (ie, x-axis smaller than zero). We make use of four quadrants to represent our predicted directions visually. Points within the two green quadrants correspond to correct forecasts of the direction, while those in the red quadrants indicate incorrectly predicted increases or decreases. The legend provides the percentage of forecasts in each of these quadrants. Second, figure 1 provides insights into our ability to forecast changes in GAM. Here, the x-axis and y-axis indicate the predicted and actual change, respectively, and are in percentage points. The dashed line represents precisely correct forecasts.

The results presented in figure 1 show that the random forest performs best in predicting the correct direction of the change. Specifically, the random forest achieves accurate forecasts for the direction in 72.9% of test observations (49.6%+23.3%), a substantial improvement compared with the naive benchmark’s 37.3% (17.1%+20.2%). On examining errors, it becomes apparent that the majority of errors arise from failing to forecast decreases (eg, a false increase of 20.2% for random forest), whereas errors in predicting increases are relatively minimal (eg, a false decrease of 7% for random forest). This implies that the model rarely overlooks increases, an important finding for the application of these methods.

Concerning the forecasts of the absolute values of changes, we observe that, while they occasionally align precisely with the true values (as seen on the dashed line), such instances are limited and occur most often in the random forest forecast. Despite this, the tree-based machine learning methods outperform the benchmarks, with their forecasts overall clustered closer to the dashed line. On closer examination of the distances to this line, which indicate the absolute forecast errors, we note that the tree-based forecasts maintain a similar distance across all magnitudes of true change in GAM. This indicates that the performance is consistent for both small and large changes. Notably, even for substantial positive changes in the test set (where true GAM change ≥1%), forecasts remain relatively close to the line.

**Figure 2:**
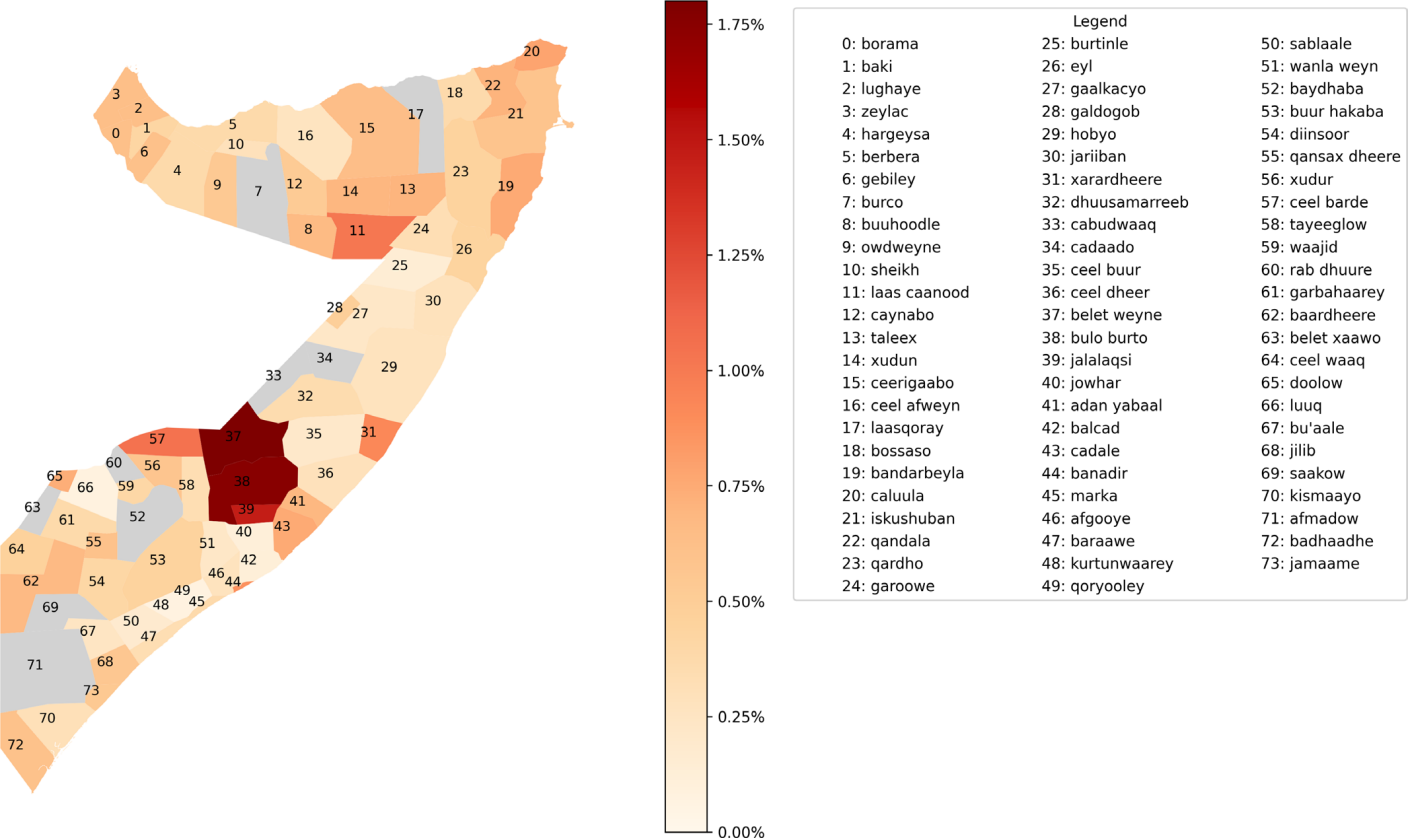
Mean absolute error (MAE) of random forest forecasts per district, in percentage points of global acute malnutrition (GAM).

Finally, we turn to the accuracy of our forecasts at the district level, and therefore we present the MAEs of our random forest forecasts in figure 2, which are aggregated by district. The errors of the random forest are exclusively presented due to its best performance. The errors are in terms of percentage points of GAM. Darker shading indicates larger errors, and districts in grey are excluded from our analyses due to data scarcity issues.

The accuracy results shown in figure 2 reveal that the model errors are mostly below 0.6% and are fairly consistent across districts, except for the districts of Belet Weyne, Bulo Burto and Jalalaqsi in the Hiiraan region. For each of these districts, there is one test set observation (the last quarter of 2020) that does not follow the broader trends captured in the data. The data report an average GAM decrease of 2.65% for these districts during this period, a significant contrast to other time periods and neighbouring regions where either smaller decreases, neutral or positive changes were observed. Hence, these few specific test set observations do not follow the trend the model expects. For more details on how the model uses different variables to make predictions, we refer to the SHapley Additive exPlanations analysis^35^ in section 5 of the online supplementary material.

## Discussion

We found rainfall, land vegetation quality, food security status, crop production and demographic factors to be associated with GAM. The majority of these associations are nonlinear. However, in interpreting these findings, it is crucial to recognise that these correlations may reflect bidirectional relationships or may be influenced by unmeasured (confounding) factors. Therefore, further research is necessary to confirm any causal relationships.

Machine learning algorithms were found to perform better than the naive and simple exponential smoothing benchmarks. Among the prediction methods tested, random forest machine learning is the most effective at accurately forecasting GAM prevalence 6 months ahead in test data for the majority of districts in Somalia. The random forest method consistently outperforms the other three methods across four performance criteria: overall MAE, accuracy in predicting the direction of change, accuracy in forecasting the magnitude of change and reliability in providing granular forecasts at the district level. However, it is important to acknowledge its limitations, particularly the poorer performance in predicting decreases, over increases.

The GAM prevalence data in this study were collected every 6 months from 2017 to 2021, giving estimates for nine distinct biannual time points. This has important implications for the robustness of outputs from the models, as the small number of time points may distort or otherwise decrease accuracy and precision of models. It also means that if changes in observed or unobserved factors impacted most or all districts in the country, there is a particularly notable possibility of confounding bias. Moreover, many of the collected potential factors, such as disease-related variables, exhibited missing observations exceeding 60%, which could have provided valuable insights if the data were more complete. We conjecture that the presence of errors and outliers in datasets, particularly in food prices and GAM, may have impacted the accuracy of forecasts.

Nonetheless, few countries with a high prevalence of GAM produce prevalence data as frequently as the FSNAU initiative in Somalia, or at district-level resolution. As such, exploring the potential application of statistical and machine learning methods to assess the potential for better predictive methods in Somalia using this data is considered well warranted.

To enhance the quality of the presented analyses, we see potential in exploring additional predictive factors beyond those included, for example, using data directly from Google Earth engine or similar, rather than using secondary sources as is used in this study.

While improving these data issues are of primary concern to improve the quality of the analysis, we also see some secondary future opportunities. Given some of the limitations posed by data scarcity, alternative modelling approaches such as Bayesian machine learning techniques may warrant consideration as they have been shown to perform well in data sparse environments.^36 37^

The findings presented are used at the WFP to further investigate methods for improving GAM estimates.

## Conclusion

Acute malnutrition is a pressing global health problem affecting an estimated 45 million children under 5 years of age worldwide and accounting for a minimum of 800 000 child deaths each year.^3 5 38^ Currently, initiatives aimed at addressing the condition are hindered by the unreliability of estimates and forecasts of GAM caseloads. Responding to the need to improve these estimates, this study explores the application of statistical and machine learning methods which could support identification of factors associated with GAM and forecast future prevalence. Our approach is applied using a large and recent set of publicly accessible data for the country of Somalia but holds the potential to be applied to other countries.

Analysis of statistical relationships, based on simple and multiple linear and non-parametric regressions, confirms that GAM prevalence is both time- and region-dependent in Somalia. Furthermore, we identify several factors associated with GAM prevalence, including the prevalence of GAM from 6 months prior, rainfall, NDVI scores, population in IPC phase 3 or higher, crop diversity, crop production, district population count and district density. Importantly, we establish that the majority of these identified factors exhibit a nonlinear relationship with GAM prevalence, a characteristic that is often overlooked when directly relying on parametric statistical analysis.

In response to the need to anticipate the number, timing and locations of GAM cases, we apply tree-based machine learning to generate spatiotemporal forecasts for the districts in Somalia. Results show effectiveness in forecasting directions and changes in GAM prevalence at the district level 6 months in advance. Compared with the naive method, which predicts only 37.3% of directions correctly, our forecasts achieve an accuracy of 72.9%. These forecasts can inform programming and targeted outreach 6 months prior, facilitating anticipatory action.

## Supplementary material

10.1136/bmjph-2024-001460 online supplemental file 1

## Data Availability

Data are available in a public, open access repository.
