# Supplementary material for: Identification of factors associated with acute malnutrition in children under 5 years and forecasting future prevalence: assessing the potential of statistical and machine learning methods
Source: BMJ Public Health. 2025 Mar 4;3(1):e001460. doi: 10.1136/bmjph-2024-001460 (PMC11883882; doi:10.1136/bmjph-2024-001460)
Supplement: online supplemental file 1 [file bmjph-3-1-s001.pdf]

Supplementary material for publication:

Reusken, M., Coffey, C., Cruijssen, F., Melenberg, B., and van Wanrooij, C. (2024). Identification of factors associated with acute malnutrition in children under five years and forecasting future prevalence: Assessing the potential of statistical and machine learning methods. *BMJ Public Health.*

1 Guide to access online data used in our study

ACLED. The Armed Conflict Location and Event Data provides access to conflict data. This database is freely accessible after registration on the web page using the Data Export Tool (https://acleddata.com/data-export-tool/). The data contains information on each individual event, presenting the category of the event (e.g., armed battles, explosions, protests and riots) and the location of the event.

OWID. Our World in Data provides data on the prevalence of COVID-19 (https://ourworldindata.org/coronavirus-source-data). This data contains the number of new COVID-19 cases and the number of new COVID-19 deaths between March 2020 and January 2022 on a national level.

FSNAU. The Food Security and Nutritional Analysis Unit created an Early Warning and Early Action (EW/EA) dashboard (https://dashboard.fsnau.org/). The data behind this dashboard is publicly available when registering to the website. The data in the dashboard is grouped by month. The following variables are retrieved from the EW/EA: rainfall, Normalized Difference Vegetation Index (NDVI) scores, food prices, new admissions of acutely malnourished children in treatment programmes, insecurity – incidents and fatalities, spread of disease and displacements. In addition, the production of crops in metric tons on a district level are stored in a different online database published by the FSNAU (https://crops.fsnau.org/). The first observation in this database dates back to 1995. The production amounts are available for cowpea, ground nuts, maize, onion, pepper, rice, sesame, sorghum, tomato and watermelon. The prevalence of GAM data is also initially collected by the FSNAU, and contains total population, under-five population, total SAM, MAM and GAM cases.

IPC. The Integrated Food Security Phase Classification (IPC) is a method which integrates several sources of evidence into a single score on a five-point scale where one indicates no food insecurity, and five indicates a famine. It is created for around 30 countries and classifies each region and district within a country. Details on the method can be found on their website (https://www.ipcinfo.org). The data used in our study was extracted using the 'IPC Population Tracking Tool' on their website. The database contains the following information: time series of current IPC phase, population analyzed, the number of people in each IPC phase, the projections for the coming six months and acute malnutrition.

World Bank. The World Bank provides access to location data containing the area of the districts in square meters and the x and y coordinates of the centers of each of the districts. These values were taken from a dataset published by the World Bank in a working paper called 'Predicting Food Crises'. The data was retrieved through the World Bank Microdata portal (https://microdata.worldbank.org/index.php/catalog/3811).

2 Scatter plots of the data

The following scatter plots show the raw data for the selected variables in our study, filtered to include only the period from July 2017 to July 2021. The region names have been standardized across all datasets (e.g., 'ceel bur' and 'ceel buur' have been aligned).

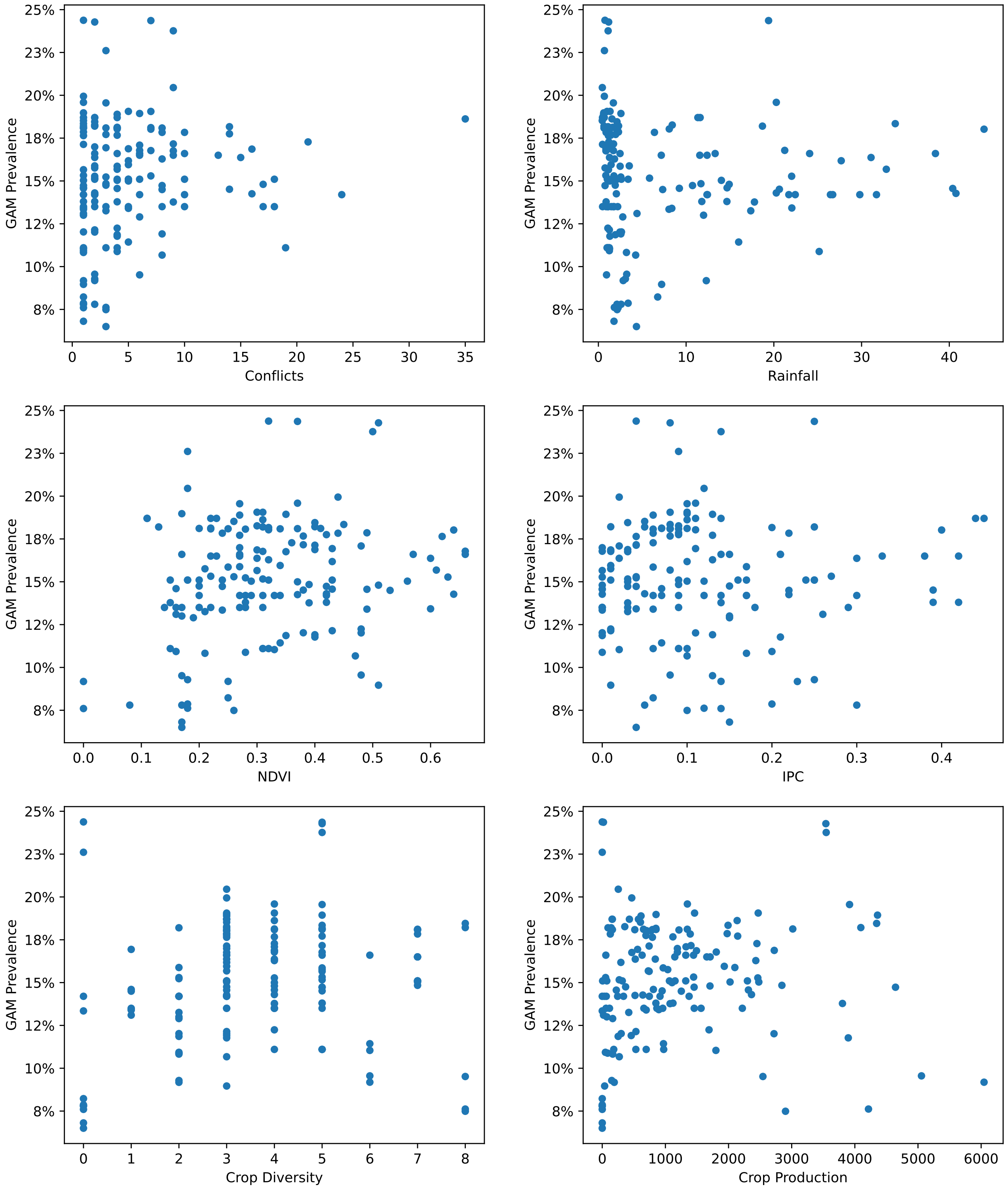

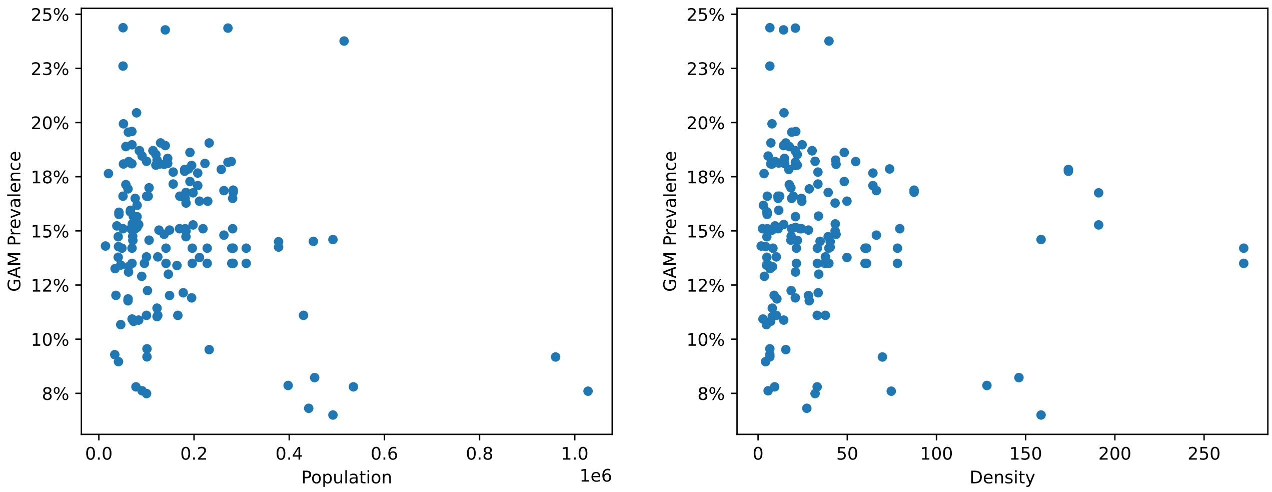

3 Code setup

We use Python 3.9 with scikit-learn for random forest and gradient boosting implementations (both in regressor configuration). For simple exponential smoothing and regression models, we employ the statsmodels implementation using default parameters.

For random forest, we conduct an exhaustive grid search over the following parameter space:

```
n_estimators: [10, 50, 100, 200]
max_depth: [1, 3, 5, 7, 9, 11, None]
min_samples_split: [2, 5, 10]
min_samples_leaf: [1, 2, 4]
max_features: [1, 'sqrt', 'log2']
bootstrap: [True, False]
criterion: ['absolute_error', 'friedman_mse', 'squared_error'].
```

Using 4-fold cross-validation on the training dataset, we arrive at the following optimal parameters:

```
max_features='log2'
n_estimators=100
max_depth=None
min_samples_split=10
criterion='absolute_error'
min_samples_leaf=1
bootstrap=False.
```

For the exact interpretation of each hyperparameter, please refer to the relevant scikit-learn documentation. For gradient boosting, we employ a sequential search strategy, optimizing one parameter at a time. We explore:

```
max_features: range(5, 21)
min_samples_split: [10, 15, 20, 25, 30, 35, 40, 45, 50, 55, 60]
```

```
max_depth: [5, 6, 7, 8, None]
n_estimators: [10, 50, 100, 200, 300, 400, 500, 1000]
learning_rate: [0.05, 0.10].
```

This sequential search yields optimal parameters of:

```
max_features=12
min_samples_split=40
max_depth=7
n_estimators=100
learning_rate=0.05.
```

For the exact interpretation of each hyperparameter, please refer to the scikit-learn documentation.

4 Detailed results of statistical relationship analysis

This document contains detailed results for the statistical analyses performed to establish the correlation (Section 4.1) and nature (Section 4.2) of the relationship between GAM and the following input variables: GAM $6lag$, conflicts, conflicts $1lag$, conflicts $3sum$, covid, covid $1lag$, covid $3sum$, rainfall, rainfall $1lag$, rainfall $3sum$, NDVI, NDVI $1lag$, NDVI $3avg$, IPC, IPC $6lag$, crop diversity, crop diversity $6lag$, crop production, crop production $6lag$, population, population $6lag$, density and density $6lag$, where $1lag$ indicates a one-month lag, $6lag$ a six-month lag, $3sum$ a sum of the preceding three months and $3avg$ an average of the preceding three months. See Table 2 in the main paper for a description of these variables. Section 4.3 presents the results of multiple linear regression analyses used to identify factors among the set of input variables that are associated with GAM.

4.1 Simple linear regression

The first step of the statistical relationship analysis concerns determining the significance of the relationship between GAM and each input variable separately. We present the significance (p value) and the coefficient (Coef.) of regressing each input variable on GAM in Table 1. Additionally, the final column ('Result') of this table provides the conclusion that can be drawn from each regression regarding the statistical association. Based on the p value, this result can fall into four categories:

'very high':	$p \leq 0.01$;
'high':	$0.01 < p \leq 0.05$;
'low':	$0.05 < p \leq 0.3$;
'very low':	$p > 0.3$.

Table 1 shows that all results of the non-dummy input variables are significantly different from zero with 95% confidence, except for rainfall $3sum$. For the effect of months on GAM, we find a significant relationship with

p values below 5% for July, August and January. The simple linear regressions for the remaining months are not significant. Additionally, some of the dummy variables for the regions yield insignificant results.

These results are used for findings presented in the column 'Simple linear regression' in Table 3 in the main paper.

Table 1. Simple linear regression results. The result columns indicate statistical associations based on p value thresholds: 'very high' for $p \leq 0.01$; 'high' for $0.01 < p \leq 0.05$; 'low' for $0.05 < p \leq 0.3$; and 'very low' for $p > 0.3$.

Input variable	p value	Coef.	Result
GAM			
$6lag$	0.000	0.805	very high
Conflicts	0.000	0.160	very high
$1lag$	0.000	0.165	very high
$3sum$	0.000	0.178	very high
Rainfall	0.010	0.043	very high
$1lag$	0.038	0.035	high
$3sum$	0.052	0.034	low
NDVI	0.000	0.313	very high
$1lag$	0.000	0.311	very high
$3avg$	0.000	0.354	very high
IPC	0.000	−0.127	very high
$6lag$	0.000	−0.178	very high
Crop diversity	0.000	0.412	very high
$6lag$	0.000	0.469	very high
Crop production	0.000	0.211	very high
$6lag$	0.000	0.216	very high
Population	0.007	−0.045	very high
$6lag$	0.045	−0.037	high
Density	0.000	0.067	very high
$6lag$	0.000	0.081	very high

Input variable	p value	Coef.	Result
Months			
January	0.050	−0.119	high
February	0.119	−0.095	low
March	0.246	−0.071	low
April	0.448	−0.046	very low
May	0.720	−0.022	very low
June	0.967	0.003	very low
July	0.024	0.124	high
August	0.020	0.142	high
September	0.137	0.091	low
October	0.513	0.040	very low
November	0.857	−0.011	very low
December	0.310	−0.062	very low
Regions			
Awdal	0.000	−1.316	very high
Bakool	0.000	1.343	very high
Banadir	0.000	0.563	very high
Bari	0.000	0.302	very high
Bay	0.000	1.134	very high
Galgaduud	0.316	−0.070	very low
Gedo	0.289	0.066	low
Hiraan	0.000	0.576	very high
Juba Dhexe	0.001	0.285	very high
Juba Hoose	0.522	0.047	very low
Mudug	0.115	0.104	low
Nugaal	0.075	0.150	low
Sanaag	0.006	−0.232	very high
Shabelle Dhexe	0.172	−0.100	low
Shabelle Hoose	0.000	0.684	very high
Sool	0.000	−0.841	very high
Togdheer	0.000	−1.821	very high
Woqooyi Galbeed	0.000	−1.663	very high

4.2 Simple nonparametric regression

This section presents the statistical analyses conducted to draw conclusions about the nature of the relationship between GAM and every input variable. We present outcomes of regressing GAM on each of the input variables

separately, using (and comparing) both linear and nonparametric regressions.

Figure 3 presents the regression of GAM on its six months lagged value, and indicates that there is likely some autoregression. Moreover, since the red curve and red confidence interval are very close to the blue curve, the linear autoregression is close to the nonparametric variant, except for very low values of GAM. We hence conclude that the nature of the relationship between GAM and GAM 6*lag* is linear.

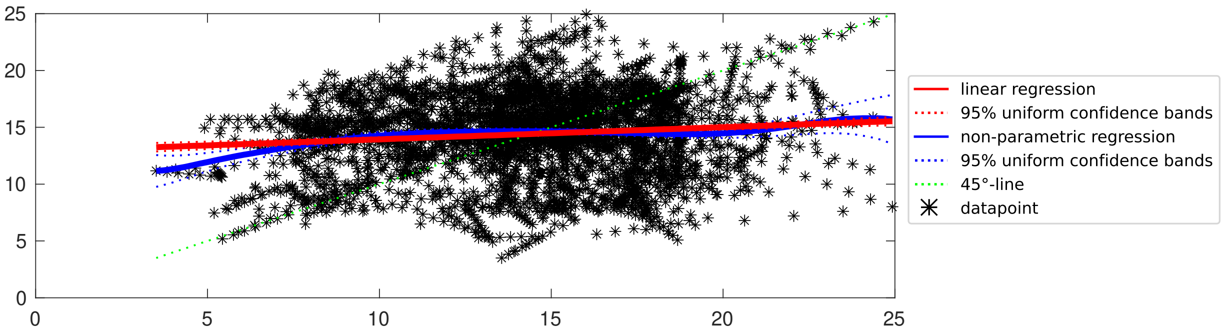

Figure 3. The regression estimates of GAM (y-axis) on GAM 6*lag* (on x-axis).

We next turn to the nonparametric regressions of GAM on each of the other input variables. For this, we show the regressions for each of the regions. We make use of the numbering of the regions as presented in Table 2.

Table 2. Numbering of regions.

	Region
0 :	Awdal
1 :	Bakool
2 :	Banadir
3 :	Bari
4 :	Bay
5 :	Galgaduud
6 :	Gedo
7 :	Hiraan
8 :	Juba Dhexe
9 :	Juba Hoose
10 :	Mudug
11 :	Nugaal
12 :	Sanaag
13 :	Shabelle Dhexe
14 :	Shabelle Hoose
15 :	Sool
16 :	Togdheer
17 :	Woqooyi Galbeed

The detailed results for each of these regions and for every input variable (except the six months lagged value, GAM 6*lag*, as this result is already presented in Figure 3) are included in Appendix A. We carefully analyzed each of these figures by comparing the nonparametric results with the linear results. When the linear regression confidence interval is within the nonparametric interval, we classify that the link between GAM and the input variable is linear with 95% confidence.[1] In the scenario that the linear regression does not fit inside the non-parametric 95% confidence band, we categorize the outcome as nonlinear. Table 3 summarizes this comparative analysis for all relationships presented in Appendix A.

[1]Since nonparametric regression converges slower than linear regression, the formal rule based on asymptotics is to check whether the linear regression curve fits within the nonparametric confidence bands. However, in our finite sample case we apply a stricter version of this rule by requiring the linear confidence intervals, rather than just the linear curve, to be within the nonparametric confidence bands.

The table is organized into three sections: the left section outlines the input variables, the middle section details individual results for each region, and the right section presents aggregate results. The '1' in black signifies that linear regression intervals *strictly* fall inside the nonparametric bounds. The '1' in red indicates that the linear regression confidence interval *generally* lies within the nonparametric confidence bands, albeit with less strict conformity in the tails. This leniency is useful to prevent false rejection of linearity, especially when dealing with a small number of observations in the tails. The aggregate results in the right section of the table contains two subcolumns: the first indicating the sum of regions where linearity is identified and the second expressing this sum as a percentage of the total number of regions in the dataset. This percentage serves as a basis for concluding linearity; specifically, if a linear relationship is identified in over 50% of the regions, the percentage is displayed in bold font and the outcome is categorized as linear. In all other instances, the outcome is categorized as nonlinear.

Table 3. Summary results of comparing the confidence bands of the linear and nonparametric regression for every input variable and every region.

	Regions																			
Input variable	0	1	2	3	4	5	6	7	8	9	10	11	12	13	14	15	16	17	Totals	
Conflicts	1	1	1					1	1	1	1	1							8	44%
1*lag*	1		1						1	1	1	1			1				7	39%
3*sum*				1											1				2	11%
Rainfall	1			1						1			1			1	1	1	7	39%
1*lag*	1									1			1			1	1	1	6	33%
3*sum*	1									1				1			1	1	5	28%
NDVI		1		1	1		1	1	1	1	1		1	1	1		1		12	**67%**
1*lag*		1	1	1				1	1	1			1	1	1		1		10	**56%**
3*avg*	1	1	1	1	1	1	1	1	1	1	1		1	1	1		1		15	**83%**
IPC									1	1		1							3	17%
6*lag*											1	1						1	3	17%
Crop diversity	1		x	x		1				1	1	x	x			x	1	1	6	46%
6*lag*	1	1	x	x		1				1	1	x	x			x		1	6	46%
Crop production	1		x	x		1		1	1		1	x	x			x			5	38%
6*lag*	1		x	x		1			1		1	x	x			x			4	31%
Population	1		1													1	1	1	5	28%
6*lag*	1		1			1											1	1	5	28%
Density	1	1	1										1			1		1	6	33%
6*lag*	1		1	1														1	4	22%

1 = Linear regression confidence interval is strictly within nonparametric confidence bands.
1 = Linear regression confidence interval is generally within the nonparametric confidence bands, being less strict on the tails.
x = No data available.

In conclusion, the outcomes presented in Table 3 reveal that only variable 'NDVI' is characterized by a linear relationship with GAM, while relationships with conflicts, rainfall, IPC, crop diversity, crop production, population and density are characterized as nonlinear. These results are used for conclusions presented in the column 'Simple nonparametric regression' in Table 3 in the main paper.

4.3 Multiple linear regression

In the final step of the statistical relationship analysis, our focus is on finding the input variables that collectively impact GAM. We investigate the presence of a statistically significant relationship using multiple linear

regression models. Tables 5–8 present these results for three models linking GAM to the set of input variables. The models test for different variants of the input variables: (Model 1) the original variants, (Model 2) the derived variants $6lag$ and $1lag$ and (Model 3) the derived variants $6lag$, $3sum$ and $3avg$. These three models include dummy variables to account for the effect of months and region[2]. We calculated the standard errors imposing homoskedasticity and zero autocorrelation (cf. Tables 5 and 6) but also allowing for heteroskedasticity and autocorrelation (cf. Tables 7 and 8). In the latter case we made use of the Newey-West standard errors. The outcomes are quite comparable.

In addition, Table 4 presents the F-test results for the significance of including months and regions. We make use of F-tests to determine if the dummy variables for months collectively, as well as the dummy variables for regions collectively, contribute significantly to explaining the variance in GAM prevalence. Therefore, we specifically examine two F-tests: one that considers the dummy variables for months, and another that considers the dummy variables for regions. Upon analyzing these results, we observe that the two F-tests are both jointly significant. Hence, we can conclude that the inclusion of months and regions in the model significantly improves the fit compared to a model without these dummy variables.

Furthermore, the results in Tables 4, 5 and 7 identify statistical associations based on the following p value thresholds:

$$\begin{aligned} &\text{`very high':} && p \leq 0.01; \\ &\text{`high':} && 0.01 < p \leq 0.05; \\ &\text{`low':} && 0.05 < p \leq 0.3; \\ &\text{`very low':} && p > 0.3. \end{aligned}$$

The findings presented in the 'Result' columns of Tables 4 and 5 are presented in the column 'Multiple linear regression' in Table 3 in the main paper.

Table 4. F-test results for a joint linear hypothesis, for months and regions.

	Months	Regions
Degrees of freedom	11	17
F-test statistic	1.8734	261.9724
p value	0.0381	0.0000
Result	high	very high

[2] We also tested the variant with district dummies, which yielded partially different results but did not change the implications of the analysis; that is, no different inputs would be selected for the forecasting analysis.

Table 5. Regression results with GAM prevalence as the target variable. The coefficients (Coef.) and standard errors (Std. Err.) are presented, along with the significance levels (p values) and the result for statistical associations based on p value thresholds: 'very high' for $p \leq 0.01$; 'high' for $0.01 < p \leq 0.05$; 'low' for $0.05 < p \leq 0.3$; and 'very low' for $p > 0.3$. Note that the p values for Crop production $6lag$ and Density $6lag$ between Model 2 and Model 3 result in a different p-category, with the 'Result' column reporting the best performance.

	Model 1			Model 2			Model 3			
	Coef.	Std. Err.	p value	Coef.	Std Err.	p value	Coef.	Std. Err.	p value	Result
Constant	−0.723	0.077	0.000	−0.628	0.073	0.000	−0.653	0.073	0.000	very high
GAM *6lag*	0.432	0.016	0.000	0.421	0.016	0.000	0.427	0.016	0.000	very high
Conflicts	−0.013	0.022	0.558							very low
Rainfall	0.021	0.010	0.038							high
NDVI	−0.035	0.018	0.048							high
IPC	0.060	0.014	0.000							very high
Crop diversity	0.102	0.021	0.000							very high
Crop production	0.019	0.013	0.135							low
Population	−0.034	0.025	0.178							low
Density	0.372	0.566	0.511							very low
Conflicts $1lag$				−0.012	0.022	0.586				very low
Rainfall $1lag$				0.007	0.010	0.486				very low
NDVI $1lag$				−0.077	0.018	0.000				very high
IPC $6lag$				0.001	0.012	0.904	−0.008	0.012	0.542	very low
Crop diversity $6lag$				0.182	0.021	0.000	0.184	0.021	0.000	very high
Crop production $6lag$				−0.023	0.013	0.079	−0.026	0.013	0.041	high
Population $6lag$				−0.052	0.024	0.032	−0.053	0.024	0.029	high
Density $6lag$				0.915	0.509	0.072	1.088	0.509	0.033	high
Conflicts $3sum$							−0.028	0.025	0.268	low
Rainfall $3sum$							0.000	0.010	0.993	very low
NDVI $3avg$							−0.132	0.020	0.000	very high
Region dummies	yes			yes			yes			
Month dummies	yes			yes			yes			

Table 6. Regression results of the dummy variables, corresponding to the regressions presented in Table 5. The coefficients (Coef.) and standard errors (Std. Err.) are presented, along with the significance levels (p value).

	Model 1			Model 2			Model 3		
	Coef.	Std. Err.	p value	Coef.	Std Err.	p value	Coef.	Std. Err.	p value
Months									
February	0.032	0.047	0.493	−0.011	0.048	0.813	0.039	0.046	0.400
March	0.073	0.047	0.119	0.015	0.049	0.761	0.039	0.047	0.409
April	0.124	0.047	0.008	0.057	0.049	0.247	0.034	0.048	0.483
May	0.193	0.049	0.000	0.123	0.048	0.011	0.078	0.048	0.108
June	0.218	0.047	0.000	0.229	0.046	0.000	0.173	0.047	0.000
July	0.243	0.047	0.000	0.249	0.047	0.000	0.257	0.046	0.000
August	0.236	0.050	0.000	0.212	0.051	0.000	0.251	0.050	0.000
September	0.210	0.050	0.000	0.171	0.052	0.001	0.183	0.051	0.000
October	0.188	0.051	0.000	0.135	0.052	0.010	0.125	0.051	0.015
November	0.195	0.053	0.000	0.127	0.051	0.013	0.099	0.051	0.053
December	0.164	0.052	0.002	0.150	0.050	0.003	0.112	0.050	0.026
Regions									
Bakool	1.465	0.074	0.000	1.488	0.075	0.000	1.560	0.076	0.000
Banadir	−2.205	5.400	0.683	−7.652	4.959	0.123	−9.217	4.959	0.063
Bari	0.769	0.061	0.000	0.751	0.062	0.000	0.745	0.062	0.000
Bay	1.355	0.083	0.000	1.316	0.084	0.000	1.427	0.087	0.000
Galgaduud	0.503	0.063	0.000	0.508	0.063	0.000	0.541	0.063	0.000
Gedo	0.477	0.072	0.000	0.423	0.073	0.000	0.488	0.074	0.000
Hiraan	0.589	0.101	0.000	0.423	0.100	0.000	0.494	0.101	0.000
Juba Dhexe	0.756	0.081	0.000	0.781	0.081	0.000	0.908	0.085	0.000
Juba Hoose	0.671	0.076	0.000	0.739	0.076	0.000	0.882	0.081	0.000
Mudug	0.602	0.061	0.000	0.609	0.061	0.000	0.624	0.061	0.000
Nugaal	0.625	0.070	0.000	0.605	0.070	0.000	0.602	0.070	0.000
Sanaag	0.385	0.068	0.000	0.402	0.068	0.000	0.403	0.068	0.000
Shabelle Dhexe	0.645	0.070	0.000	0.601	0.070	0.000	0.669	0.071	0.000
Shabelle Hoose	1.146	0.072	0.000	1.148	0.071	0.000	1.259	0.062	0.000
Sool	−0.064	0.062	0.306	−0.011	0.062	0.855	0.000	0.062	0.997
Togdheer	−0.310	0.065	0.000	−0.258	0.064	0.000	−0.227	0.064	0.000
Woqooyi Galbeed	−0.214	0.071	0.003	−0.182	0.070	0.010	−0.182	0.070	0.010

Table 7. Regression results using Newey-West standard errors. Note that the p values for Crop production $6lag$ for Model 2 and Model 3 result in a different p-category, with the 'Result' column reporting the best performance. As compared to the regression results in Table 5, the only difference in the Result column is found for Population $6lag$, for which a very high statistical association is found.

	Model 1			Model 2			Model 3			
	Coef.	Std. Err.	p value	Coef.	Std Err.	p value	Coef.	Std. Err.	p value	Result
Constant	−0.723	0.081	0.000	−0.628	0.078	0.000	−0.653	0.079	0.000	very high
GAM *6lag*	0.432	0.020	0.000	0.421	0.021	0.000	0.428	0.021	0.000	very high
Conflicts	−0.013	0.018	0.458							very low
Rainfall	0.021	0.009	0.021							high
NDVI	−0.035	0.017	0.046							high
IPC	0.060	0.018	0.001							very high
Crop diversity	0.103	0.023	0.000							very high
Crop production	0.019	0.016	0.233							low
Population	−0.034	0.019	0.074							low
Density	0.372	0.464	0.423							very low
Conflicts $1lag$				−0.012	0.018	0.496				very low
Rainfall $1lag$				0.007	0.010	0.500				very low
NDVI $1lag$				−0.077	0.019	0.000				very high
IPC $6lag$				0.001	0.016	0.924	−0.008	0.016	0.627	very low
Crop diversity $6lag$				0.182	0.023	0.000	0.184	0.023	0.000	very high
Crop production $6lag$				−0.023	0.013	0.087	−0.026	0.013	0.043	high
Population $6lag$				−0.052	0.018	0.005	−0.053	0.018	0.004	very high
Density $6lag$				0.915	0.409	0.025	1.088	0.432	0.012	high
Conflicts $3sum$							−0.028	0.020	0.166	low
Rainfall $3sum$							0.000	0.012	0.994	very low
NDVI $3avg$							−0.132	0.021	0.000	very high
Region dummies	yes			yes			yes			
Month dummies	yes			yes			yes			

Table 8. Regression results using Newey-West standard errors of the dummy variables, corresponding to the regressions presented in Table 7.

	Model 1			Model 2			Model 3		
	Coef.	Std. Err.	p value	Coef.	Std. Err.	p value	Coef.	Std. Err.	p value
Months									
February	0.032	0.056	0.566	−0.011	0.056	0.839	0.039	0.055	0.483
March	0.073	0.053	0.167	0.015	0.054	0.781	0.039	0.052	0.456
April	0.124	0.052	0.018	0.057	0.054	0.285	0.034	0.052	0.516
May	0.193	0.055	0.000	0.123	0.054	0.023	0.078	0.054	0.151
June	0.218	0.059	0.000	0.229	0.057	0.000	0.173	0.057	0.002
July	0.243	0.065	0.000	0.249	0.064	0.000	0.257	0.063	0.000
August	0.236	0.066	0.000	0.212	0.066	0.001	0.251	0.065	0.000
September	0.210	0.060	0.000	0.171	0.060	0.004	0.183	0.059	0.002
October	0.188	0.056	0.001	0.135	0.057	0.017	0.125	0.056	0.024
November	0.195	0.056	0.001	0.127	0.055	0.022	0.099	0.055	0.074
December	0.164	0.060	0.007	0.150	0.058	0.010	0.112	0.058	0.055
Regions									
Bakool	1.465	0.097	0.000	1.488	0.095	0.000	1.560	0.099	0.000
Banadir	−2.205	4.406	0.617	−7.652	3.970	0.054	-9.217	4.197	0.028
Bari	0.769	0.078	0.000	0.751	0.076	0.000	0.745	0.077	0.000
Bay	1.355	0.108	0.000	1.316	0.103	0.000	1.427	0.109	0.000
Galgaduud	0.503	0.073	0.000	0.508	0.071	0.000	0.541	0.073	0.000
Gedo	0.477	0.092	0.000	0.423	0.086	0.000	0.488	0.087	0.000
Hiraan	0.589	0.130	0.000	0.423	0.126	0.001	0.494	0.127	0.000
Juba Dhexe	0.756	0.090	0.000	0.781	0.086	0.000	0.908	0.093	0.000
Juba Hoose	0.671	0.086	0.000	0.739	0.083	0.000	0.882	0.090	0.000
Mudug	0.602	0.076	0.000	0.609	0.073	0.000	0.624	0.074	0.000
Nugaal	0.625	0.077	0.000	0.605	0.076	0.000	0.602	0.076	0.000
Sanaag	0.385	0.087	0.000	0.402	0.084	0.000	0.403	0.084	0.000
Shabelle Dhexe	0.645	0.080	0.000	0.601	0.076	0.000	0.669	0.080	0.000
Shabelle Hoose	1.146	0.083	0.000	1.148	0.079	0.000	1.259	0.085	0.000
Sool	−0.064	0.076	0.402	−0.011	0.075	0.880	0.000	0.076	0.997
Togdheer	−0.310	0.067	0.000	−0.258	0.068	0.000	−0.227	0.068	0.001
Woqooyi Galbeed	−0.214	0.064	0.001	0.182	0.063	0.004	−0.182	0.064	0.004

5 SHAP analysis of the random forest model

To better understand the driving factors behind our model's predictions, we conducted a SHAP (SHapley Additive exPlanations) analysis on the tuned random forest model used in this study. SHAP values provide a game-theoretical approach to explaining the importance of each feature and their contribution to individual predictions. It is important for the interpretation of these results to note that our model is trained to predict absolute levels of GAM rather than first differences, with the differencing performed post-prediction. SHAP dependence plots are shown for normalized (standardized) variables.

5.1 Feature importance

Figure 4 presents the SHAP summary plots, which rank features by their absolute SHAP values. The analysis reveals that crop diversity (6-month lagged) and lagged absolute prevalence of GAM are the most influential predictors in our model. The strong influence of lagged prevalence highlights the state dependency in GAM. Climate indicators, specifically rainfall and NDVI (Normalized Difference Vegetation Index), demonstrate substantial importance, along with several regional dummy variables for different parts of Somalia.

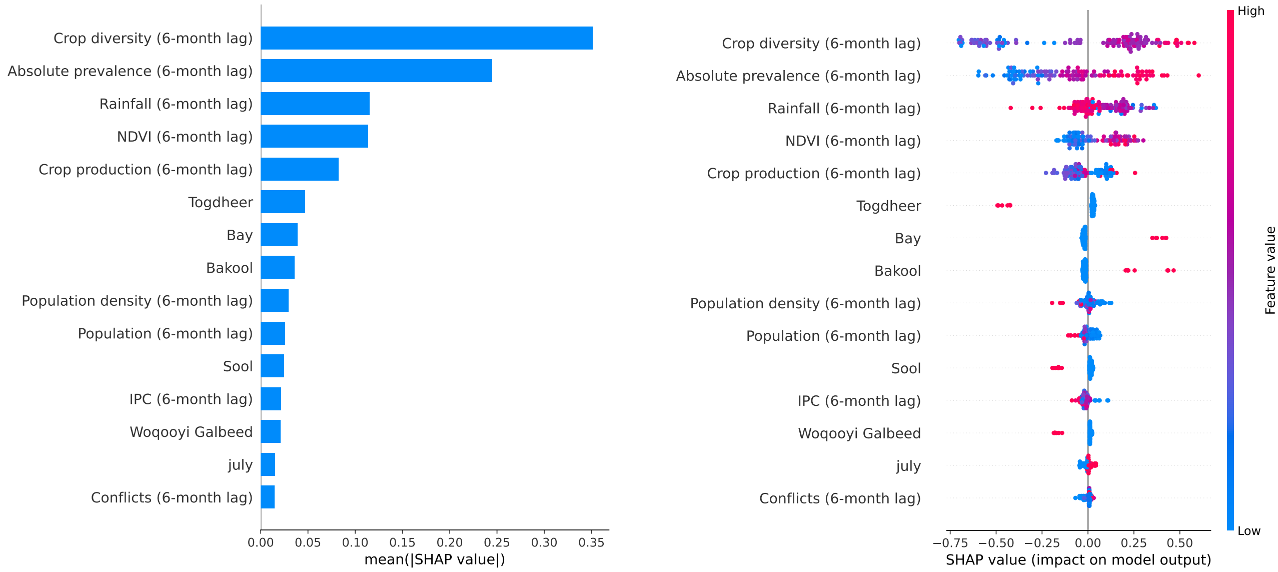

Figure 4. SHAP analysis revealing feature importance and impact direction. The left panel ranks features by their mean absolute SHAP value. The right panel shows how features impact individual predictions, where red (blue) indicates the feature contributes to increased (decreased) predictions of GAM. 'Absolute prevalence' represents the prevalence of GAM.

5.2 Feature effects

In Figures 5 and 6 we present SHAP dependence plots, revealing interesting patterns in how the model utilizes its input features. As shown in Figure 5, increased rainfall consistently leads to lower predicted levels of GAM, aligning with expectations about the relationship between precipitation and food security.

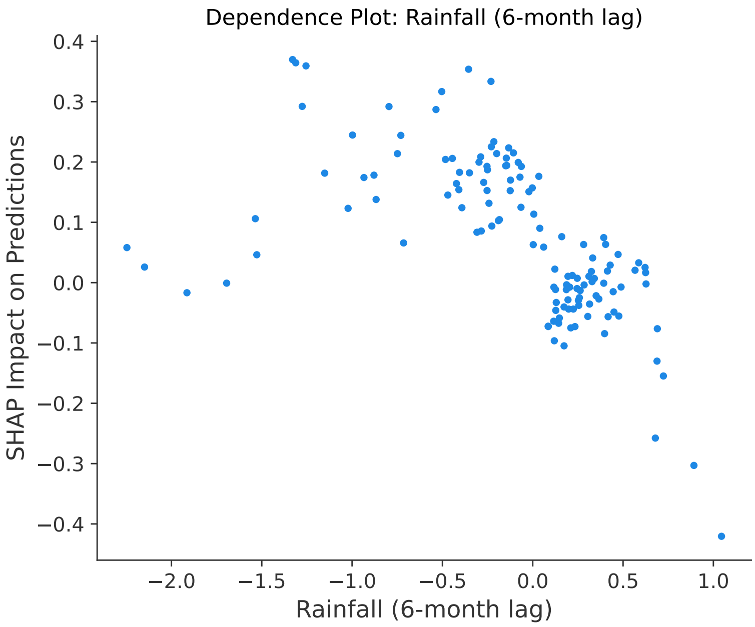

Figure 5. Dependence plot for rainfall, showing a negative relationship with predicted GAM levels.

However, the relationship between crop diversity and GAM presents a counter-intuitive pattern (Figure 6). Higher crop diversity is associated with increased predictions of GAM. This relationship likely emerges because crop diversity in our dataset inadvertently captures underlying geographic variations between northern and southern Somalia. The southern regions exhibit higher crop diversity but have historically experienced more volatility in food security outcomes, while the more arid northern regions, despite lower crop diversity, have shown more stable patterns in GAM metrics.

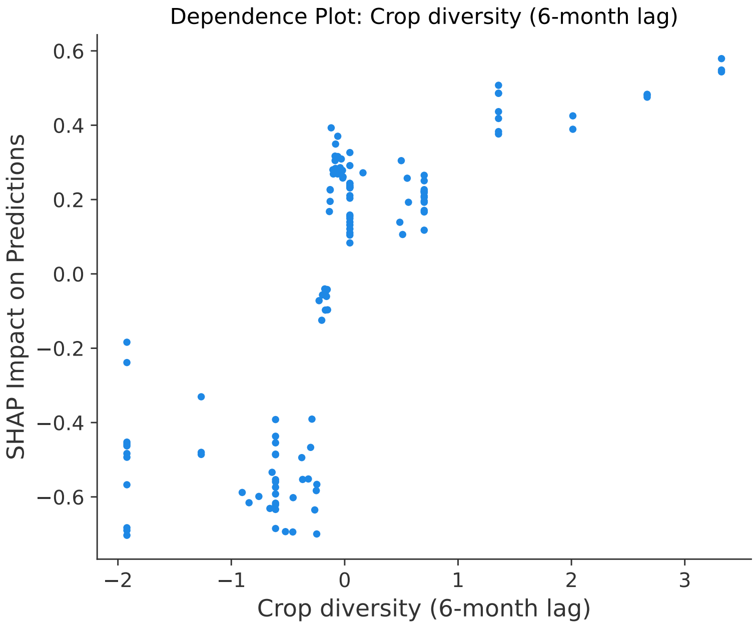

Figure 6. Dependence plot for crop diversity (6-month lag), revealing an unexpected positive relationship with predicted GAM levels due to geographic confounding.

5.3 Interpretation and limitations

The SHAP analysis suggests that our model relies heavily on spatial patterns and temporal autocorrelation in making its predictions. While climatic factors, particularly rainfall, show expected relationships with GAM, the interpretation of agricultural indicators is complicated by their correlation with underlying geographic characteristics. The model appears to have learned to use these geographic patterns as proxy indicators, which helps explain its predictive performance but complicates causal interpretation of the feature relationships. Therefore caution is needed when interpreting individual feature effects in isolation.

Furthermore, SHAP values assume feature independence, but when features are correlated, the SHAP values may not fully reflect the true contribution of each feature. While we addressed strong correlations during preprocessing by removing one of the correlated variables, some correlations in the data remain. These residual correlations could influence the SHAP values, potentially leading to biased interpretations of feature importance.

Appendix A: Simple nonparametric regression figures

The figures below depict the regression of GAM on each input variable separately, for each region (see Table 2 for the numbering of the regions). The x-axes represent the input variable reported in the title of each plot, and the y-axes represent GAM. The scales of the axes are chosen based on the regression outcomes to ensure that the regression lines and confidence bands are fully visible. This adjustment is made separately for each figure, resulting in variations in the y-axis scale from one plot to another. We make use of the legend given in Figure 7.

We analyzed each of the below figures by comparing the nonparametric results with the linear results. When the linear regression confidence interval is within the nonparametric interval, we classify that the link between GAM and the input variable is likely to be linear with 95% confidence. In the scenario that the linear regression does not fit inside the nonparametric 95% confidence band, we categorize the outcome as nonlinear. Table 3 summarizes this comparative analysis.

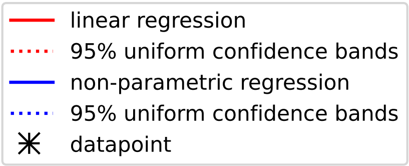

Figure 7. Legend for regression figures.

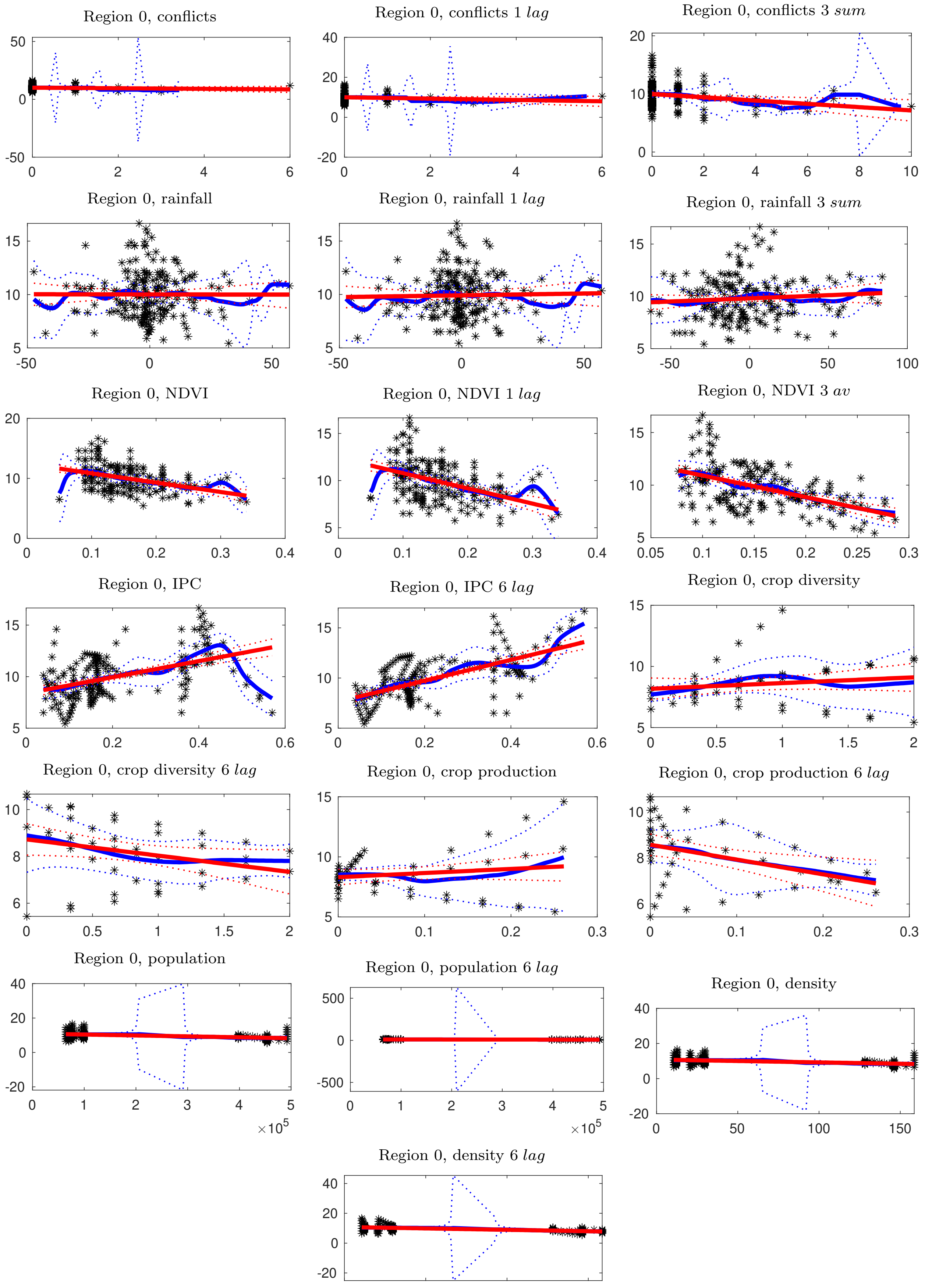
Region 0, conflicts
Region 0, conflicts 1 lag
Region 0, conflicts 3 sum
Region 0, rainfall
Region 0, rainfall 1 lag
Region 0, rainfall 3 sum
Region 0, NDVI
Region 0, NDVI 1 lag
Region 0, NDVI 3 av
Region 0, IPC
Region 0, IPC 6 lag
Region 0, crop diversity
Region 0, crop diversity 6 lag
Region 0, crop production
Region 0, crop production 6 lag
Region 0, population
Region 0, population 6 lag
Region 0, density
Region 0, density 6 lag

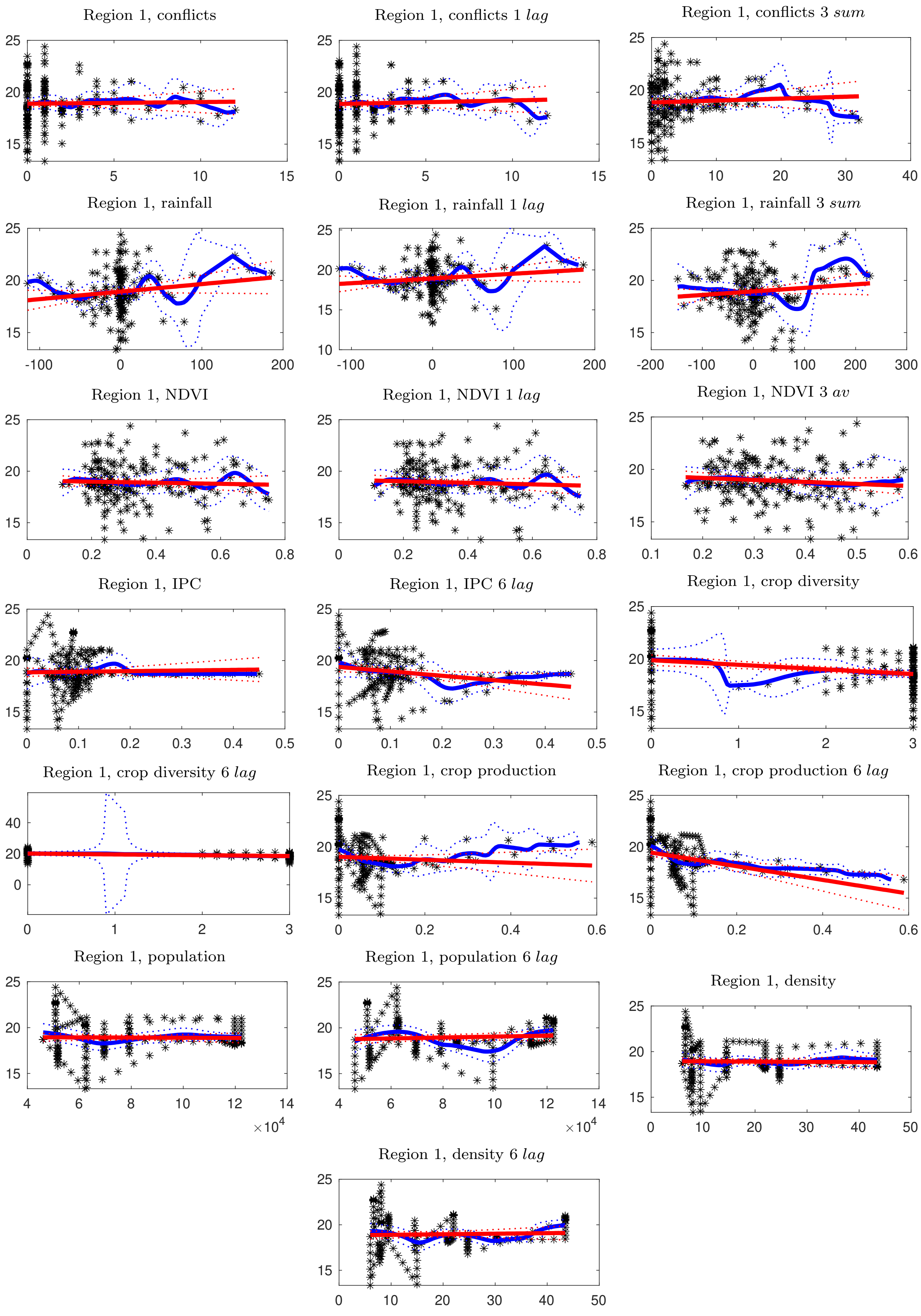
Region 1, conflicts
Region 1, conflicts 1 lag
Region 1, conflicts 3 sum
Region 1, rainfall
Region 1, rainfall 1 lag
Region 1, rainfall 3 sum
Region 1, NDVI
Region 1, NDVI 1 lag
Region 1, NDVI 3 av
Region 1, IPC
Region 1, IPC 6 lag
Region 1, crop diversity
Region 1, crop diversity 6 lag
Region 1, crop production
Region 1, crop production 6 lag
Region 1, population
Region 1, population 6 lag
Region 1, density
Region 1, density 6 lag

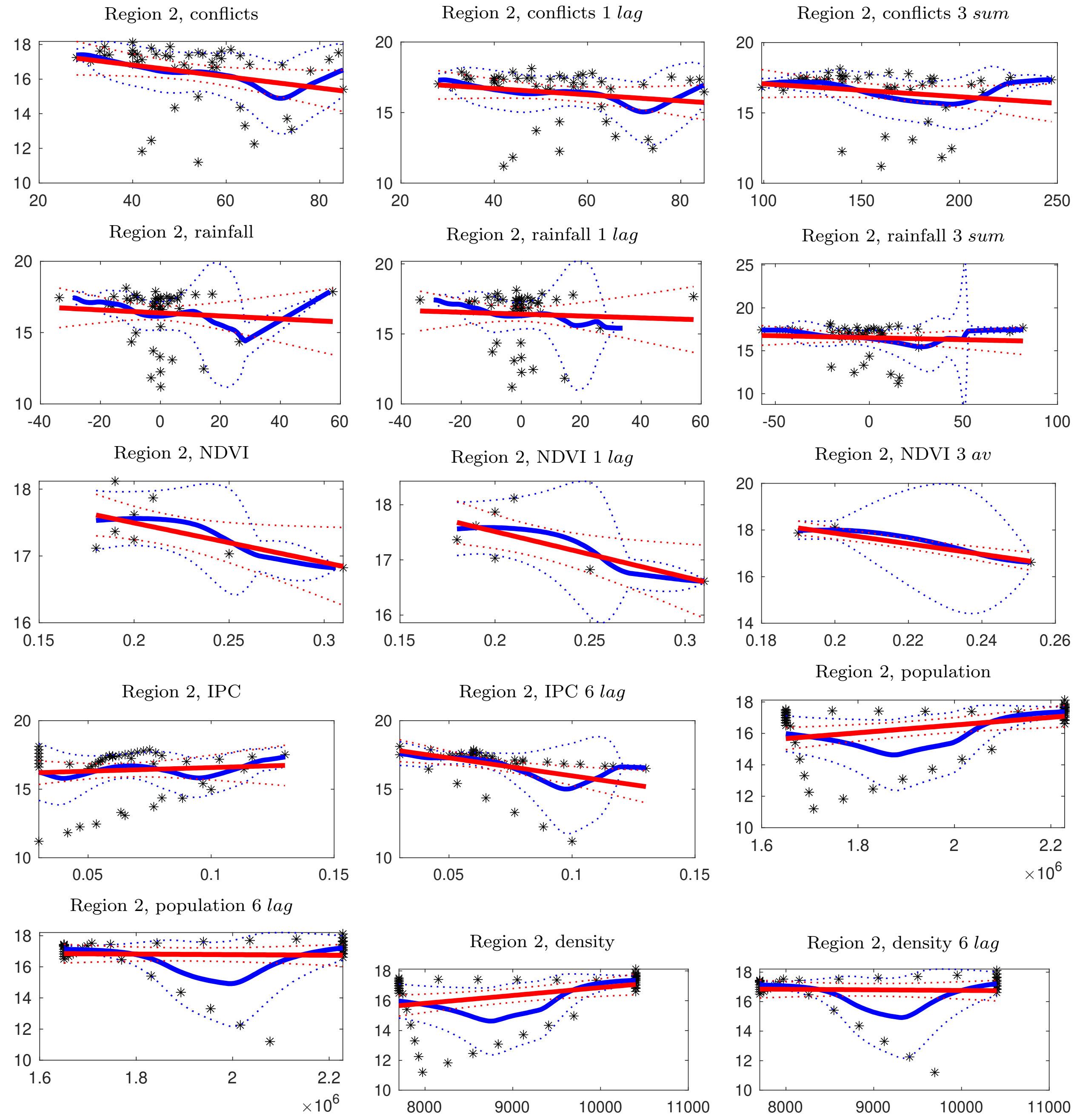

Region 2, conflicts
Region 2, conflicts 1 *lag*
Region 2, conflicts 3 *sum*
Region 2, rainfall
Region 2, rainfall 1 *lag*
Region 2, rainfall 3 *sum*
Region 2, NDVI
Region 2, NDVI 1 *lag*
Region 2, NDVI 3 *av*
Region 2, IPC
Region 2, IPC 6 *lag*
Region 2, population
$\times 10^6$
Region 2, population 6 *lag*
$\times 10^6$
Region 2, density
Region 2, density 6 *lag*

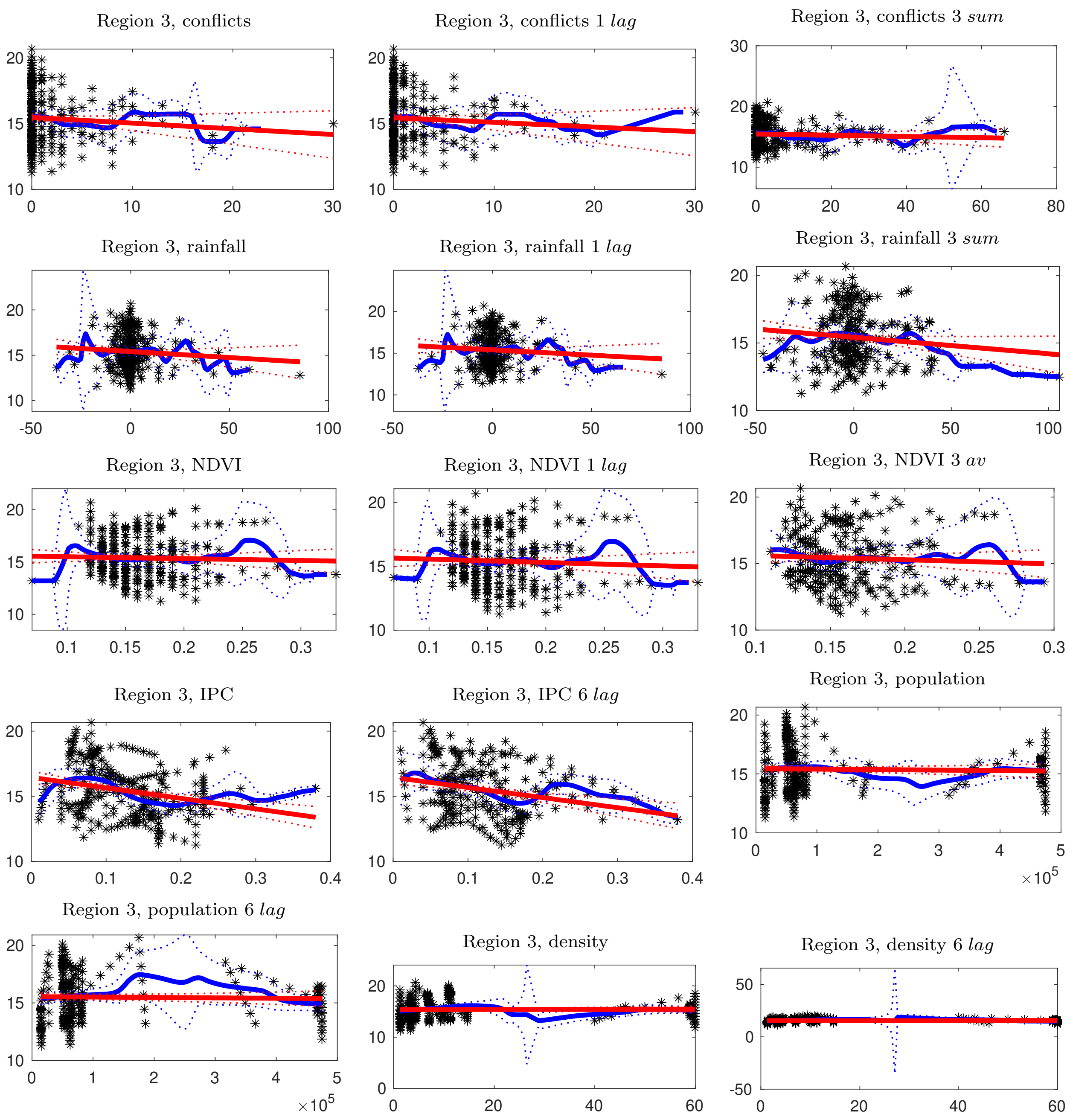
Region 3, conflicts
Region 3, conflicts 1 lag
Region 3, conflicts 3 sum
Region 3, rainfall
Region 3, rainfall 1 lag
Region 3, rainfall 3 sum
Region 3, NDVI
Region 3, NDVI 1 lag
Region 3, NDVI 3 av
Region 3, IPC
Region 3, IPC 6 lag
Region 3, population
Region 3, population 6 lag
Region 3, density
Region 3, density 6 lag
×10^5
×10^5

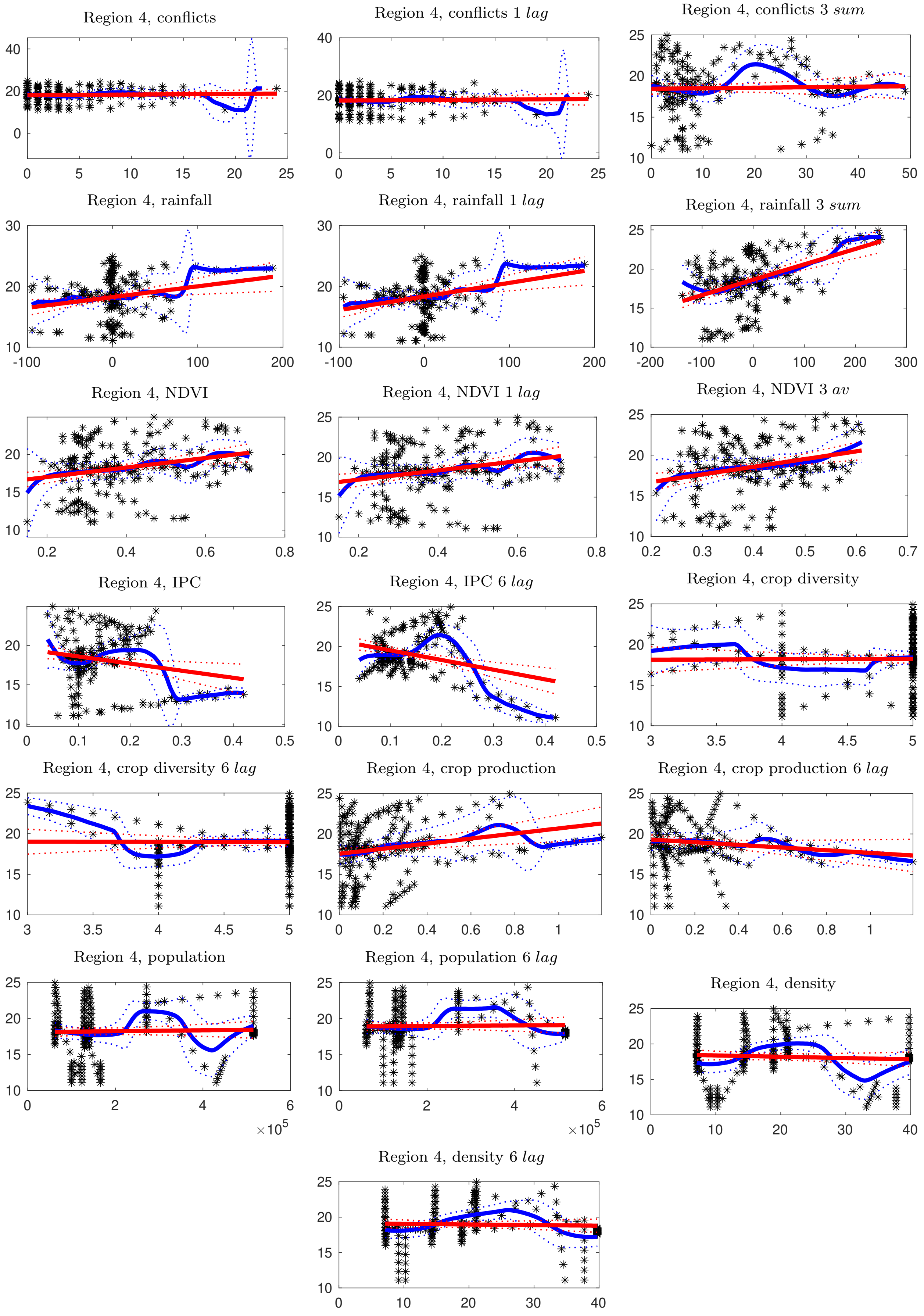
Region 4, conflicts
Region 4, conflicts 1 lag
Region 4, conflicts 3 sum
Region 4, rainfall
Region 4, rainfall 1 lag
Region 4, rainfall 3 sum
Region 4, NDVI
Region 4, NDVI 1 lag
Region 4, NDVI 3 av
Region 4, IPC
Region 4, IPC 6 lag
Region 4, crop diversity
Region 4, crop diversity 6 lag
Region 4, crop production
Region 4, crop production 6 lag
Region 4, population
Region 4, population 6 lag
Region 4, density
Region 4, density 6 lag
×10^5
×10^5

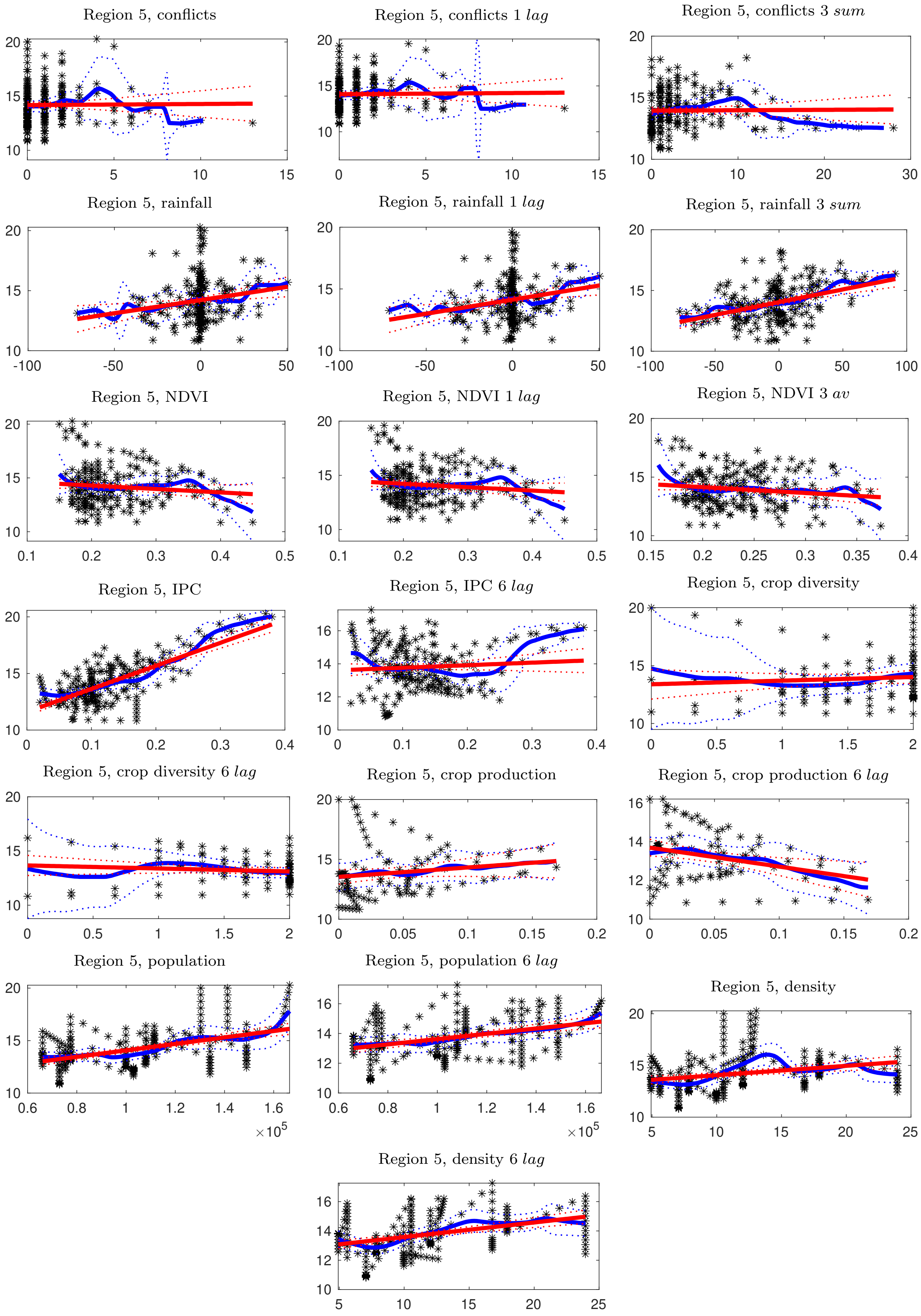
Region 5, conflicts
Region 5, conflicts 1 *lag*
Region 5, conflicts 3 *sum*
Region 5, rainfall
Region 5, rainfall 1 *lag*
Region 5, rainfall 3 *sum*
Region 5, NDVI
Region 5, NDVI 1 *lag*
Region 5, NDVI 3 *av*
Region 5, IPC
Region 5, IPC 6 *lag*
Region 5, crop diversity
Region 5, crop diversity 6 *lag*
Region 5, crop production
Region 5, crop production 6 *lag*
Region 5, population
Region 5, population 6 *lag*
Region 5, density
Region 5, density 6 *lag*

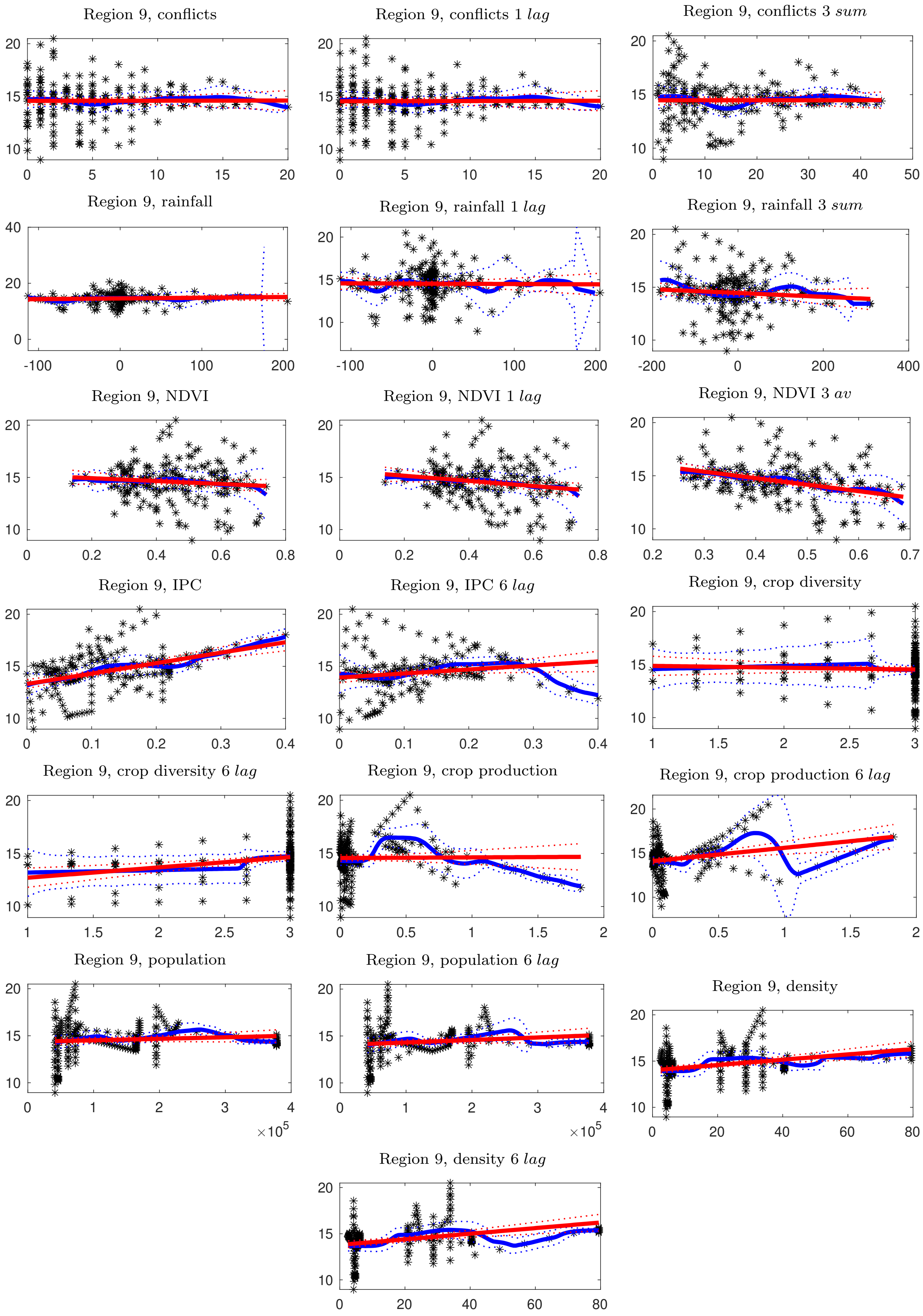
Region 9, conflicts
Region 9, conflicts 1 *lag*
Region 9, conflicts 3 *sum*
Region 9, rainfall
Region 9, rainfall 1 *lag*
Region 9, rainfall 3 *sum*
Region 9, NDVI
Region 9, NDVI 1 *lag*
Region 9, NDVI 3 *av*
Region 9, IPC
Region 9, IPC 6 *lag*
Region 9, crop diversity
Region 9, crop diversity 6 *lag*
Region 9, crop production
Region 9, crop production 6 *lag*
Region 9, population
Region 9, population 6 *lag*
Region 9, density
Region 9, density 6 *lag*
$\times 10^5$
$\times 10^5$

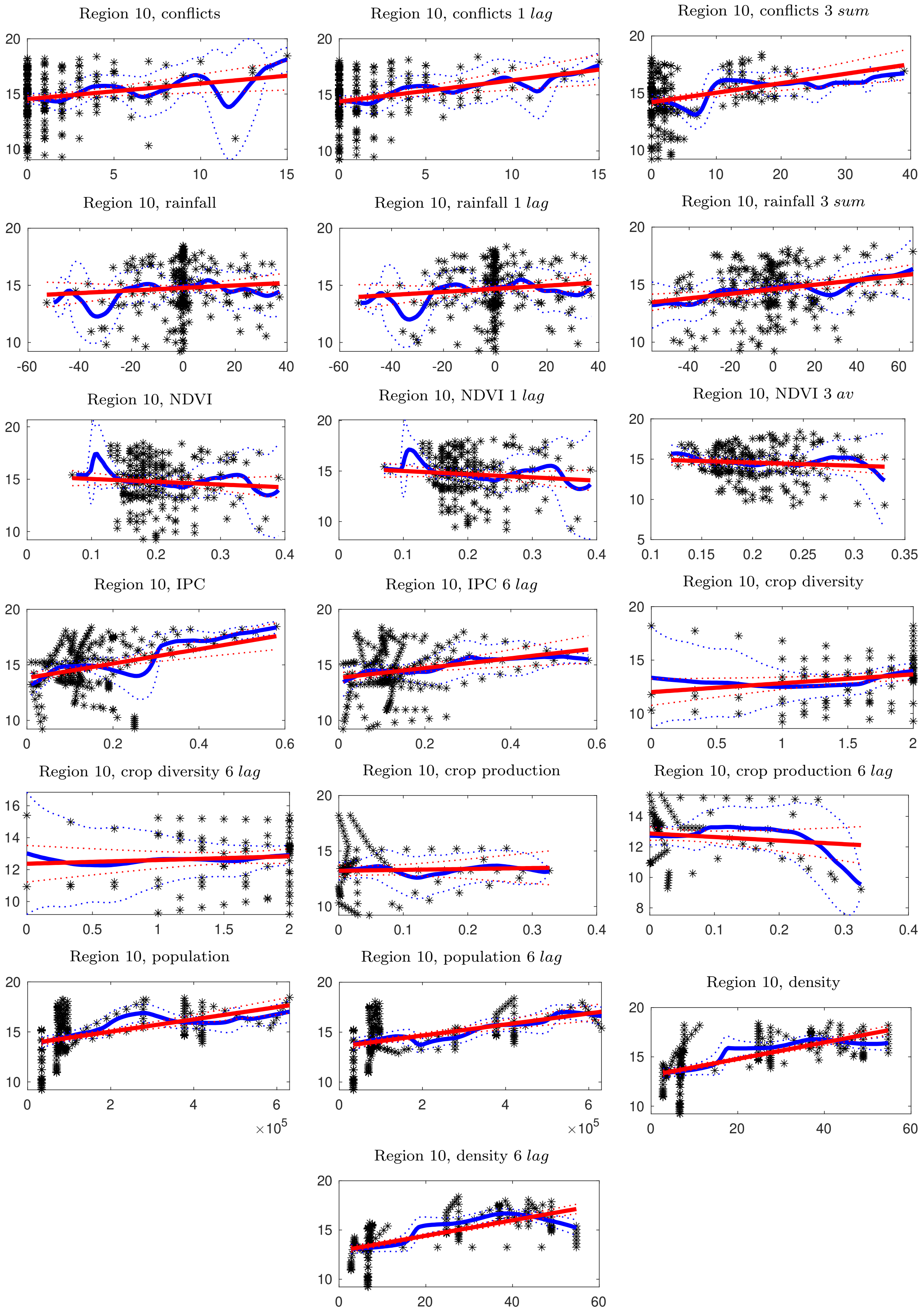
Region 10, conflicts
Region 10, conflicts 1 lag
Region 10, conflicts 3 sum
Region 10, rainfall
Region 10, rainfall 1 lag
Region 10, rainfall 3 sum
Region 10, NDVI
Region 10, NDVI 1 lag
Region 10, NDVI 3 av
Region 10, IPC
Region 10, IPC 6 lag
Region 10, crop diversity
Region 10, crop diversity 6 lag
Region 10, crop production
Region 10, crop production 6 lag
Region 10, population
Region 10, population 6 lag
Region 10, density
Region 10, density 6 lag
×10^5
×10^5

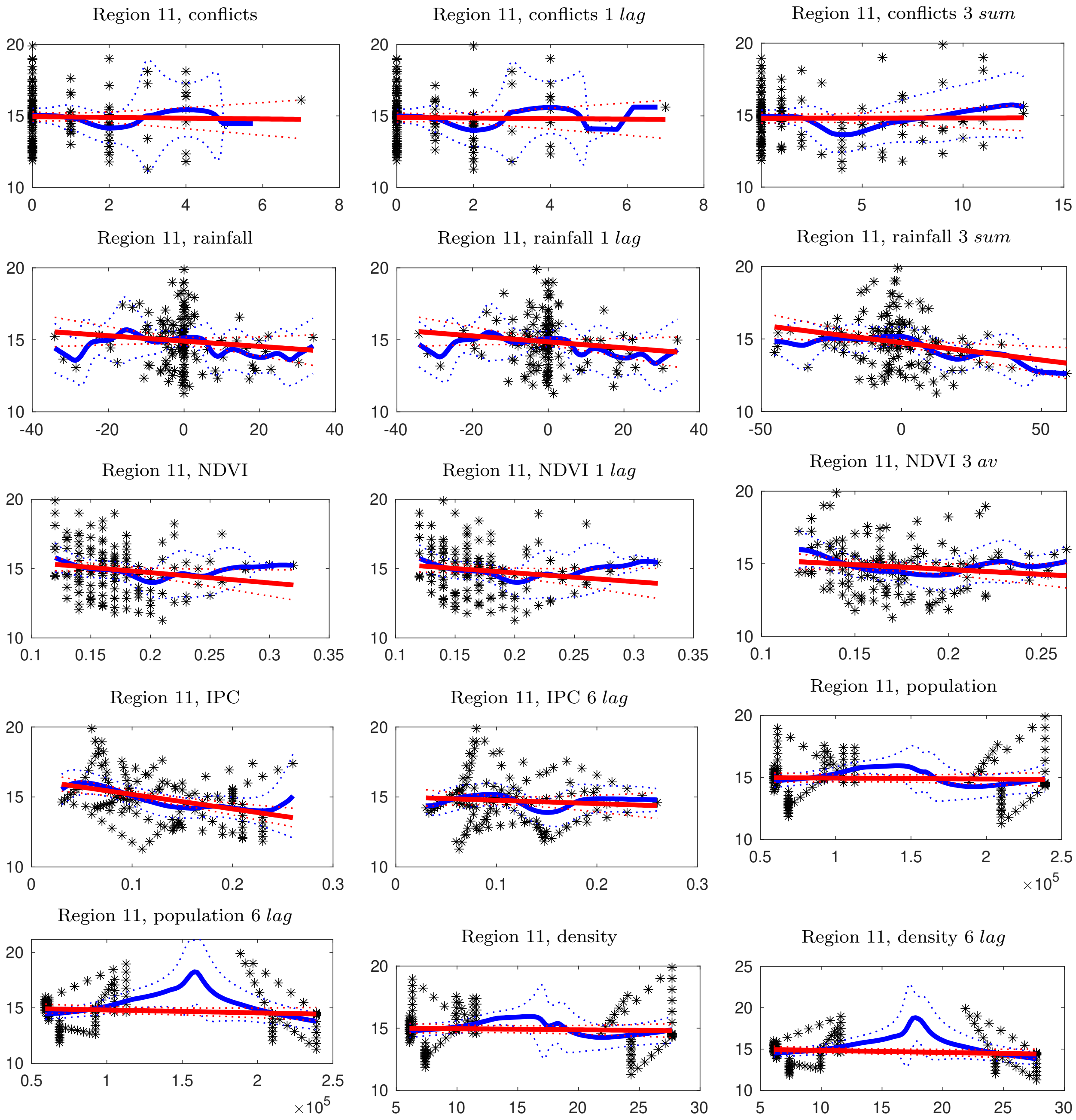
Region 11, conflicts
Region 11, conflicts 1 *lag*
Region 11, conflicts 3 *sum*
Region 11, rainfall
Region 11, rainfall 1 *lag*
Region 11, rainfall 3 *sum*
Region 11, NDVI
Region 11, NDVI 1 *lag*
Region 11, NDVI 3 *av*
Region 11, IPC
Region 11, IPC 6 *lag*
Region 11, population
$\times 10^5$
Region 11, population 6 *lag*
$\times 10^5$
Region 11, density
Region 11, density 6 *lag*

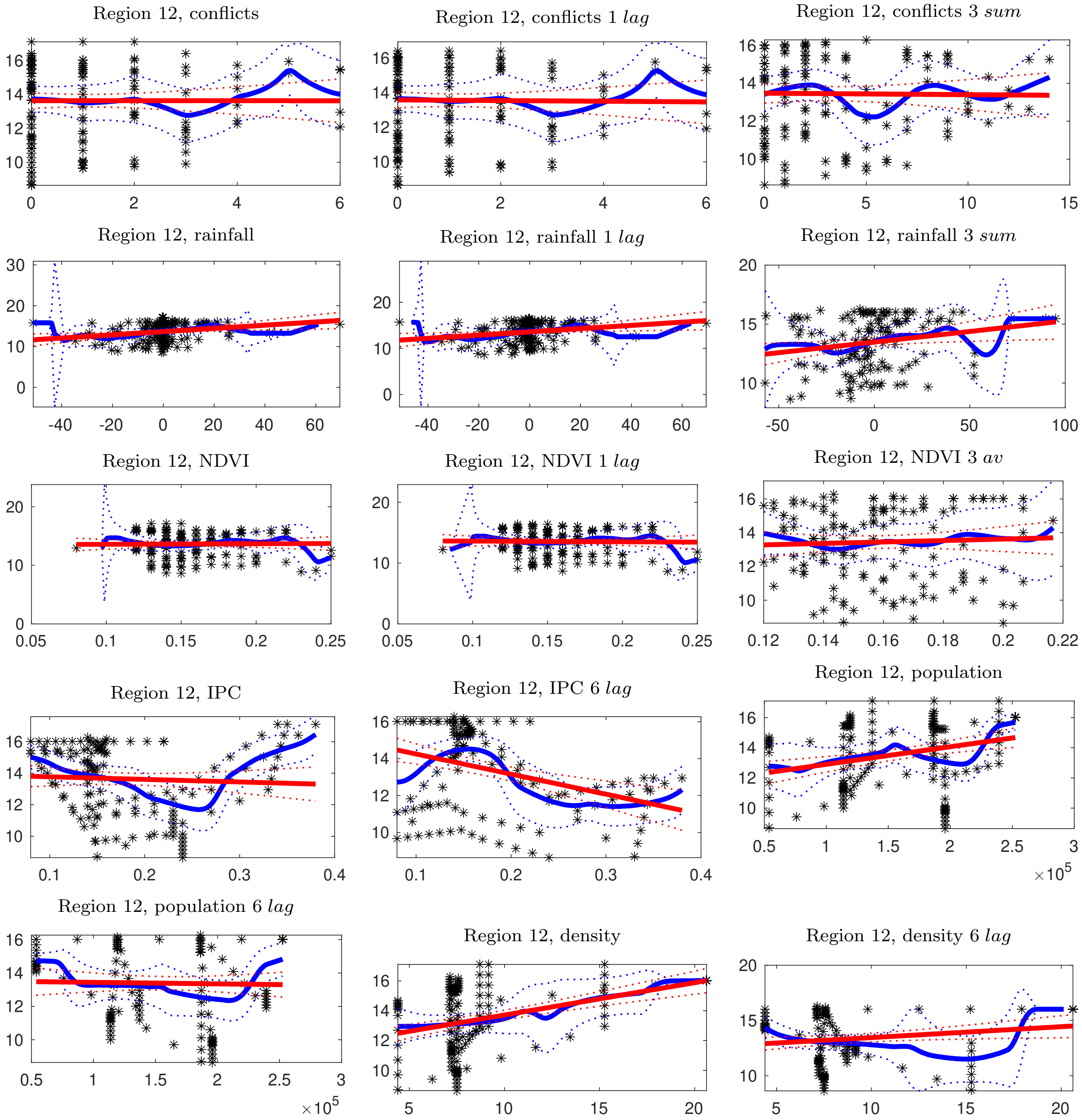
Region 12, conflicts
Region 12, conflicts 1 *lag*
Region 12, conflicts 3 *sum*
Region 12, rainfall
Region 12, rainfall 1 *lag*
Region 12, rainfall 3 *sum*
Region 12, NDVI
Region 12, NDVI 1 *lag*
Region 12, NDVI 3 *av*
Region 12, IPC
Region 12, IPC 6 *lag*
Region 12, population
$\times 10^5$
Region 12, population 6 *lag*
$\times 10^5$
Region 12, density
Region 12, density 6 *lag*

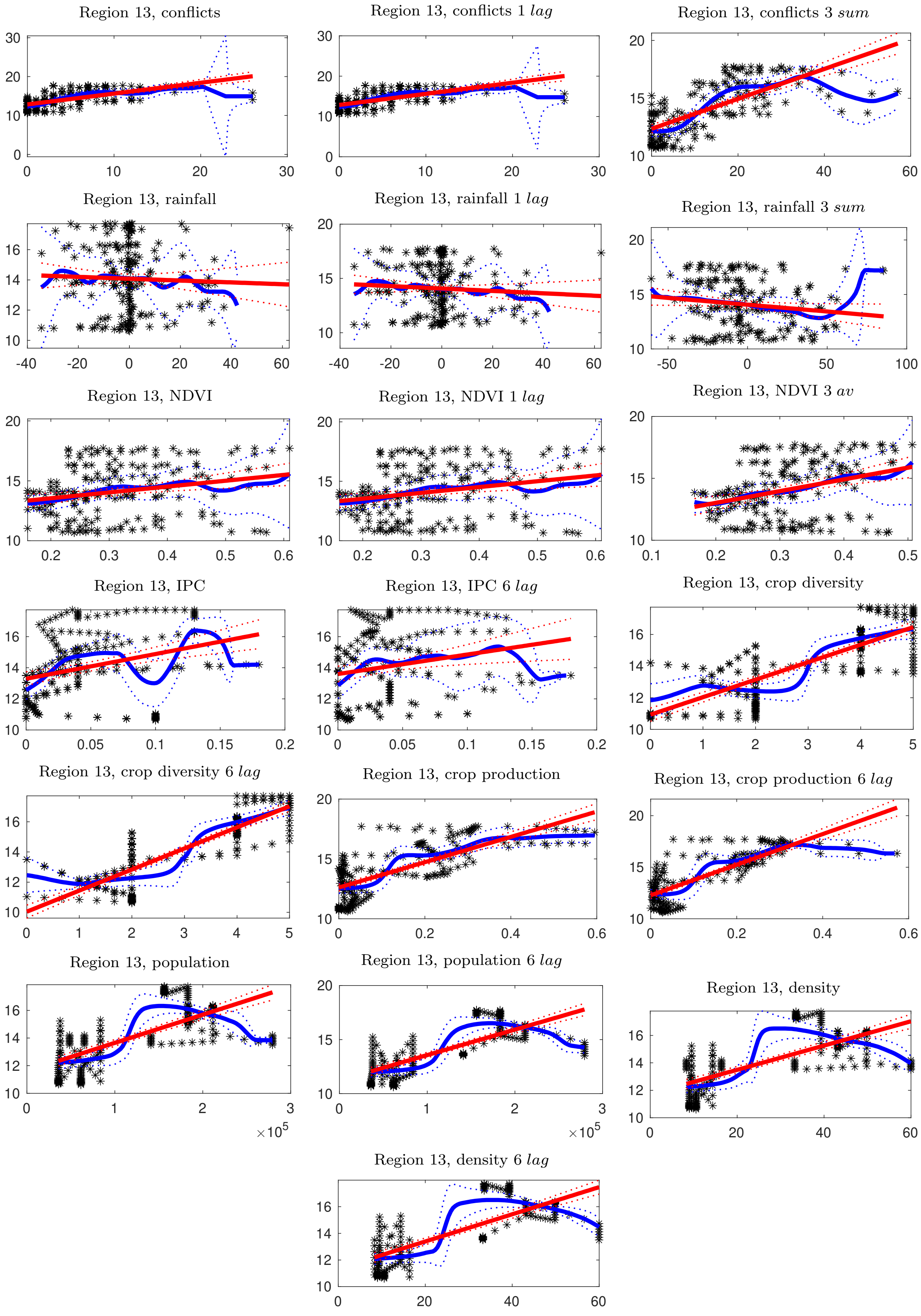

Region 13, conflicts
Region 13, conflicts 1 *lag*
Region 13, conflicts 3 *sum*
Region 13, rainfall
Region 13, rainfall 1 *lag*
Region 13, rainfall 3 *sum*
Region 13, NDVI
Region 13, NDVI 1 *lag*
Region 13, NDVI 3 *av*
Region 13, IPC
Region 13, IPC 6 *lag*
Region 13, crop diversity
Region 13, crop diversity 6 *lag*
Region 13, crop production
Region 13, crop production 6 *lag*
Region 13, population
$\times 10^5$
Region 13, population 6 *lag*
$\times 10^5$
Region 13, density
Region 13, density 6 *lag*

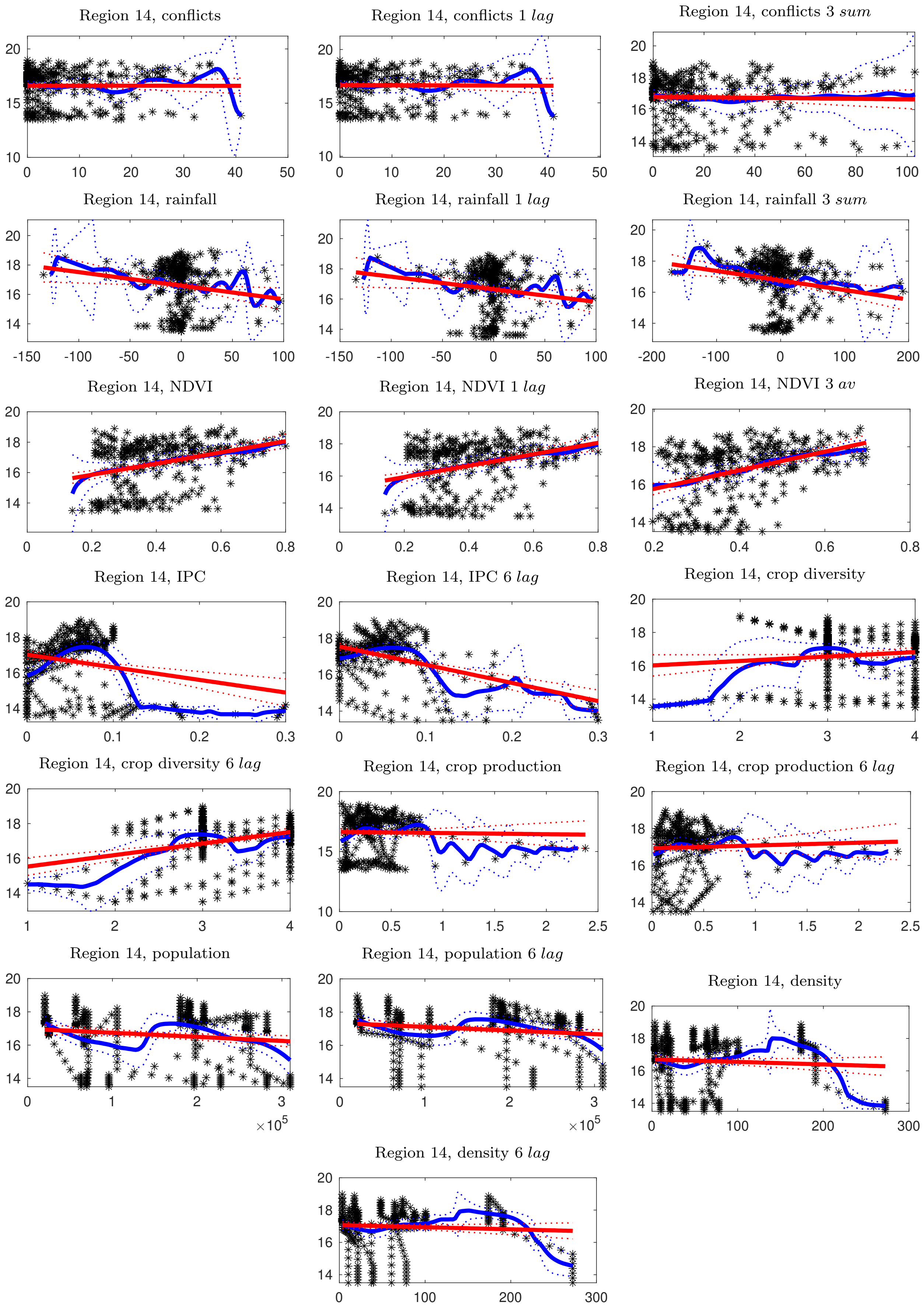
Region 14, conflicts
Region 14, conflicts 1 *lag*
Region 14, conflicts 3 *sum*
Region 14, rainfall
Region 14, rainfall 1 *lag*
Region 14, rainfall 3 *sum*
Region 14, NDVI
Region 14, NDVI 1 *lag*
Region 14, NDVI 3 *av*
Region 14, IPC
Region 14, IPC 6 *lag*
Region 14, crop diversity
Region 14, crop diversity 6 *lag*
Region 14, crop production
Region 14, crop production 6 *lag*
Region 14, population
$\times 10^5$
Region 14, population 6 *lag*
$\times 10^5$
Region 14, density
Region 14, density 6 *lag*

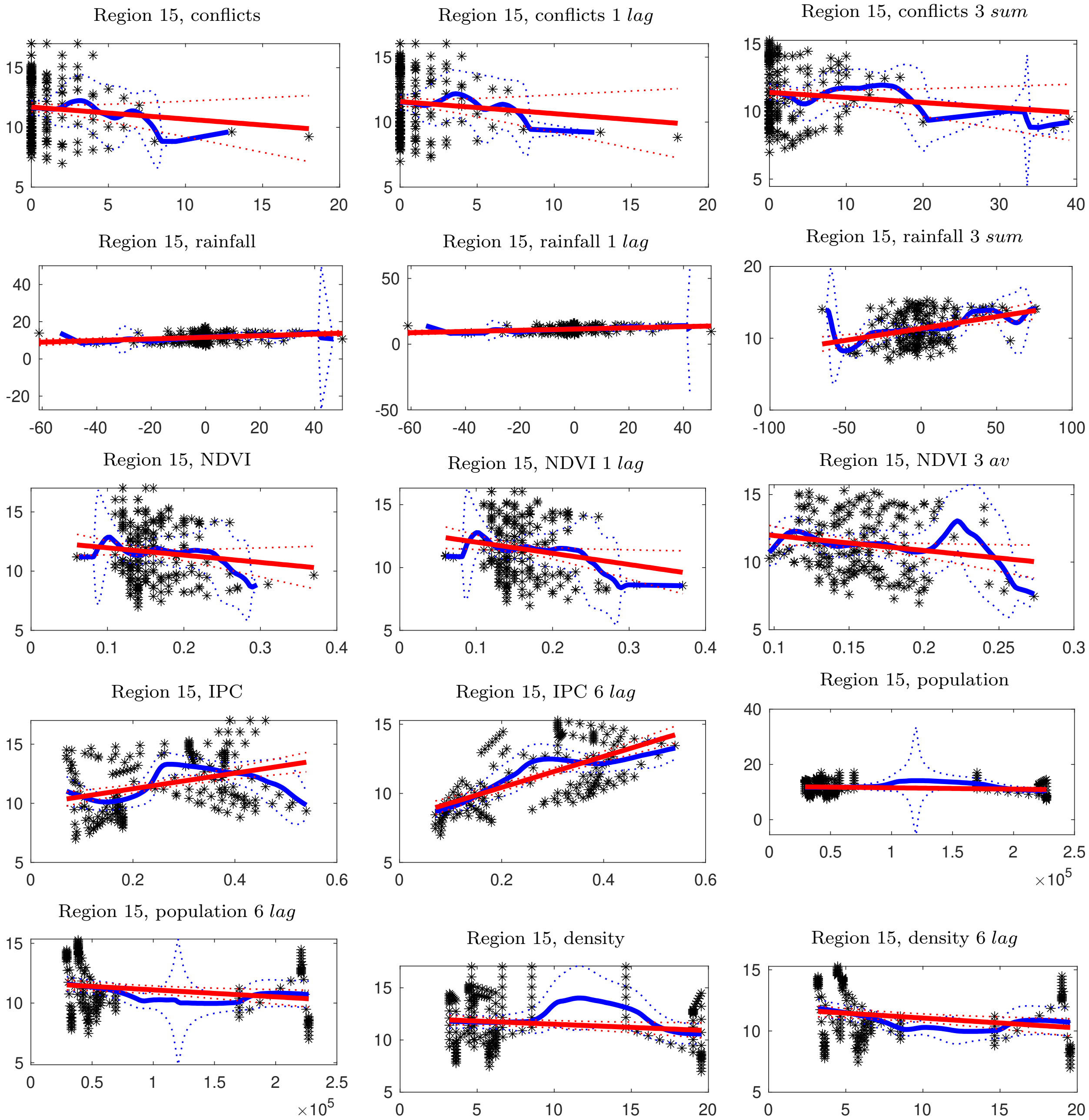
Region 15, conflicts
Region 15, conflicts 1 lag
Region 15, conflicts 3 sum
Region 15, rainfall
Region 15, rainfall 1 lag
Region 15, rainfall 3 sum
Region 15, NDVI
Region 15, NDVI 1 lag
Region 15, NDVI 3 av
Region 15, IPC
Region 15, IPC 6 lag
Region 15, population
Region 15, population 6 lag
Region 15, density
Region 15, density 6 lag

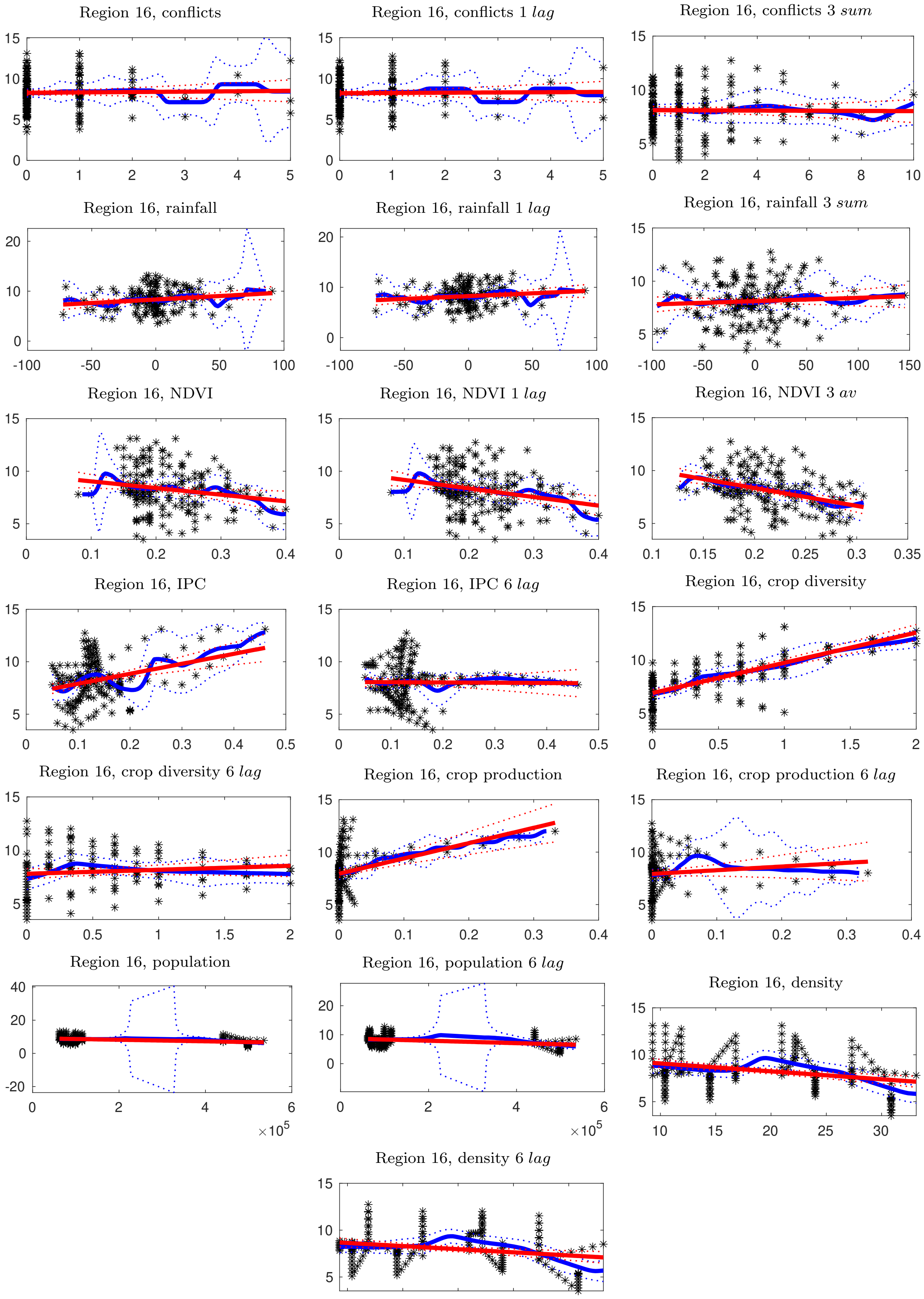
Region 16, conflicts
Region 16, conflicts 1 *lag*
Region 16, conflicts 3 *sum*
Region 16, rainfall
Region 16, rainfall 1 *lag*
Region 16, rainfall 3 *sum*
Region 16, NDVI
Region 16, NDVI 1 *lag*
Region 16, NDVI 3 *av*
Region 16, IPC
Region 16, IPC 6 *lag*
Region 16, crop diversity
Region 16, crop diversity 6 *lag*
Region 16, crop production
Region 16, crop production 6 *lag*
Region 16, population
Region 16, population 6 *lag*
Region 16, density
Region 16, density 6 *lag*

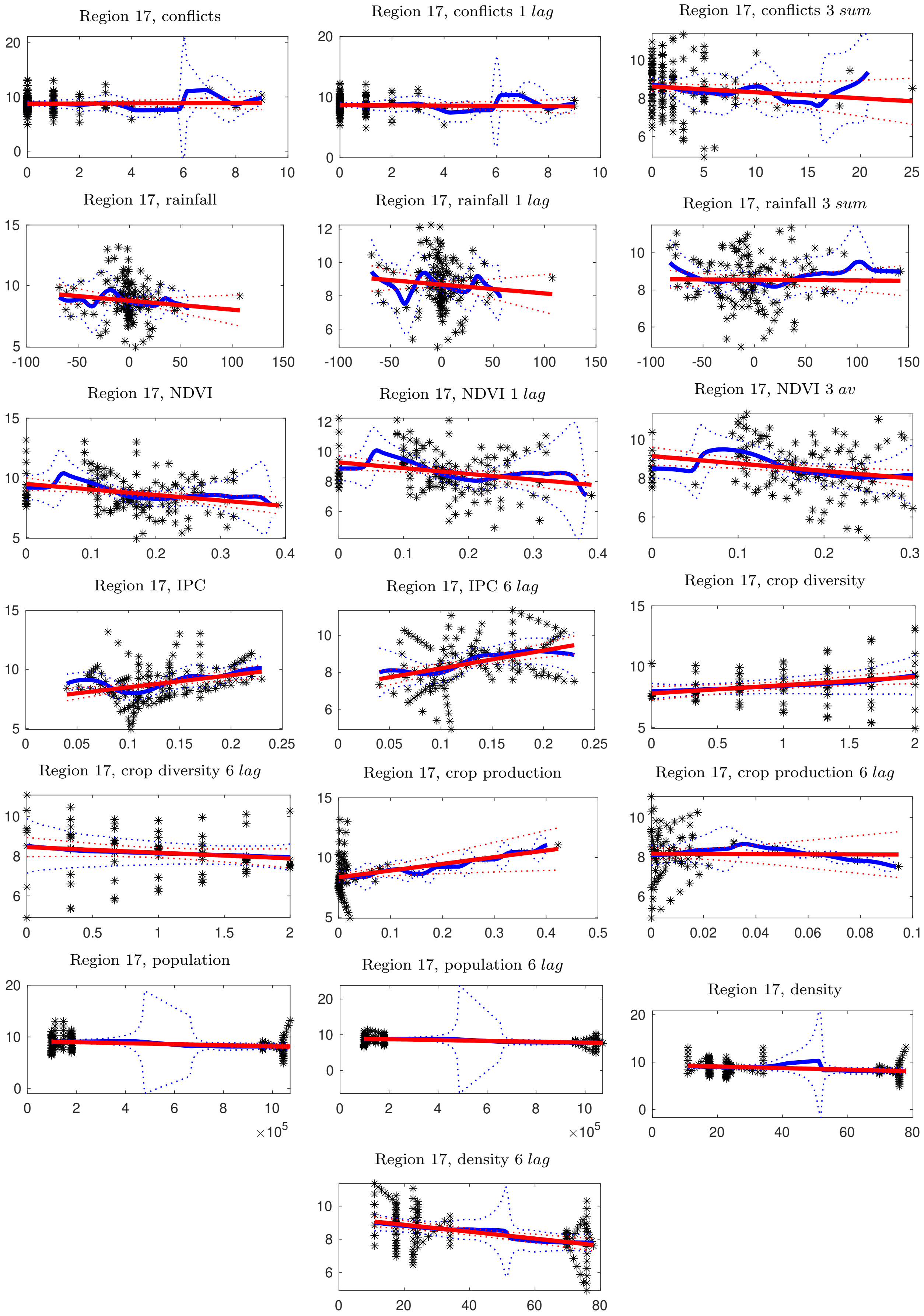
Region 17, conflicts
Region 17, conflicts 1 *lag*
Region 17, conflicts 3 *sum*
Region 17, rainfall
Region 17, rainfall 1 *lag*
Region 17, rainfall 3 *sum*
Region 17, NDVI
Region 17, NDVI 1 *lag*
Region 17, NDVI 3 *av*
Region 17, IPC
Region 17, IPC 6 *lag*
Region 17, crop diversity
Region 17, crop diversity 6 *lag*
Region 17, crop production
Region 17, crop production 6 *lag*
Region 17, population
Region 17, population 6 *lag*
Region 17, density
Region 17, density 6 *lag*
